# Astaxanthin mitigates diabetic cardiomyopathy and nephropathy in HF/HFr/STZ diabetic rats via modulating NOX4, fractalkine, Nrf2, and AP-1 pathways

**DOI:** 10.1038/s41598-025-06263-8

**Published:** 2025-06-20

**Authors:** Nesma M.E. Abo El-Nasr, Yosra A. Hussien, Marawan Abd El-Baset, Marwa E. Shabana, Dalia O. Saleh

**Affiliations:** 1 Pharmacology Department, Medical Research and Clinical Studies Institute, National Research Centre, 33 El-Bohouth St., Dokki, P.O. 12622, Cairo, Egypt; 2 Stark Neurosciences Research Institute, Indiana University School of Medicine, Indianapolis, IN USA; 3 Department of Neurology, Indiana University School of Medicine , Indianapolis, IN USA; 4 Pathology Department, Medical Research and Clinical Studies Institute, National Research Centre, 33 El-Bohouth St., Dokki, P.O. 12622, Giza, Cairo, Egypt

**Keywords:** Astaxanthin; diabetic cardiomyopathy, Diabetic nephropathy, Oxidative stress, NOX4, Fractalkine, Nrf2, AP-1, Electrocardiography, Rats, Cell biology, Drug discovery, Molecular biology, Biomarkers, Cardiology, Diseases, Health care, Medical research, Molecular medicine

## Abstract

This study investigated the effects of astaxanthin (ASTA) on diabetic cardiomyopathy (DCM) and nephropathy (DN) in rats. Type 2 diabetes was induced through a high-fat/high-fructose (HF/HFr) diet followed by a sub-diabetogenic dose streptozotocin injection. Diabetic rats were treated with ASTA at a dose of 100 mg/kg for four weeks. Serum markers of renal and cardiac function, oxidative stress parameters, and electrocardiographic (ECG) measurements were assessed. Diabetic control rats exhibited significant impairment in renal and cardiac functions, heightened oxidative stress, and altered ECG parameters. Treatment with ASTA (100 mg/kg) markedly improved these conditions, proven by reduction in serum urea, creatinine, cardiac creatine phosphokinase-MB (CK-MB), and LDH levels. Additionally, oxidative stress markers such as MDA, GSH, SOD, and NOX4 were restored in both heart and kidney tissues. Furthermore, ASTA was able to increase the cardiac and renal Fractalkine chemokine as well as attenuate the elevated Nrf2 and AP-1. ECG abnormalities were partially reversed, with enhancements in the QTc interval and ST segment height. The histopathological examination of cardiac and renal tissues confirmed these results. Finally, the forementioned promising observations suggest that ASTA may offer therapeutic potential in mitigating DCM and DN via modulation of NOX4, Fractalkine, Nrf2, and AP-1 Pathway, warranting further research into its mechanisms and clinical applicability.

**Supplementary Information:**

The online version contains supplementary material available at 10.1038/s41598-025-06263-8.

## Introduction

Diabetes mellitus (DM) is the most prevalent chronic metabolic illness and a significant threat to global health. Diabetic patients are at an increased risk of severe complications, including microvascular issues such as retinopathy, neuropathy, and nephropathy, as well as macrovascular problems like cardiovascular disease^1^. Cardiovascular diseases (CVD) are the primary cause of morbidity and death among people with DM, and patients with diabetes have a 4-to 5-fold increased risk of developing CVD^2^. Diabetic cardiomyopathy (DCM) is a distinct clinical condition characterized by structural and functional abnormalities in the myocardium, independent of other cardiovascular risk factors such as hypertension and coronary artery disease. The development of DCM is driven by multiple interconnected mechanisms, including chronic hyperglycemia, insulin resistance, oxidative stress, inflammation, and disturbances in cellular signaling pathways. These factors culminate in impaired cardiac contractility, electrophysiological disturbances, and increased vulnerability to heart failure^3^.

Similarly, diabetic nephropathy (DN) is a common and serious complication of DM, particularly in individuals with longstanding Type 2 DM. DN is triggered by lesions in the renal tubule and glomeruli. Reduced glomerular filtration rate, glomerulosclerosis, tubulointerstitial fibrosis, and destruction to the renal tubular epithelial cells are the primary pathological features of diabetic nephropathy. Because DN has an occult onset, it is challenging to identify and diagnose at an early stage. Renal lesions are permanent, and when certain symptoms arise, they finally lead to renal failure. Thus, it is crucial to detect and treat DN as soon as possible, especially with targeted therapy^4^.

Astaxanthin (ASTA)^5^, a reddish-orange carotenoid pigment, is primarily derived from the microalga *Haematococcus pluvialis* and also found in other aquatic species like salmon, trout, lobster, krill, and shrimp. It is recognized as a stronger antioxidant than other carotenoids such as lutein, beta-carotene, canthaxanthin, and zeaxanthin, with significant safety for both rodent and human use^6^. Due to its conjugated double bond and hydroxyl group, ASTA’s antioxidant capacity is reported to be 800 times stronger than coenzyme Q10, 6000 times stronger than vitamin C, and 100 times stronger than glutathione^7^.

The exceptional antioxidant properties of ASTA have garnered attention for its role in preventing diseases like diabetes, inflammation, cancer, and heart disease, as well as improving vision. Its pharmacological properties include anti-inflammatory, anti-cancer, antioxidative, and anti-apoptotic actions, which contribute to its cardioprotective and nephroprotective effects. Moreover, the potential of ASTA in managing oxidative stress-related diseases, particularly cardiovascular and metabolic disorders, is of growing interest due to its ability to scavenge free radicals, enhance antioxidant enzyme activity, and modulate inflammatory pathways^8^.

ASTA is a promising candidate in improving cardiac function and reducing oxidative stress in animal models of heart disease. While past research has linked oxidative stress and inflammation to the early stages of CVDs, antioxidants like ASTA, which can regulate lipid and glucose metabolism, restore redox balance, and adjust inflammatory responses, may help prevent conditions such as dyslipidemia, arterial hypertension, and atherosclerosis^9^. Previous studies have demonstrated that ASTA can improve cardiac function and reduce oxidative stress in animal models of heart disease, but its specific effects in the context of DCM remain underexplored^10,11^.

In diabetic nephropathy, ASTA shows potential in protecting against kidney damage by reducing oxidative stress and inflammation. Its strong antioxidant effects help restore redox balance, limit lipid peroxidation, and stabilize cell membranes, safeguarding renal cells from hyperglycemia-induced damage. Additionally, ASTA modulates glucose metabolism, lowers ROS levels, and inhibits pro-inflammatory pathways, all of which are involved in diabetic nephropathy progression^12^. Furthermore, ASTA has been observed to enhance renal function by reducing proteinuria and glomerular damage in diabetic animal models, underscoring its potential to prevent or slow the progression of renal complications in diabetes^13^.

The specific effects of ASTA in DCM and DN remain areas requiring further exploration; therefore, this study aims to explore the therapeutic potential of ASTA in mitigating diabetic cardiomyopathy (DCM) and nephropathy (DN) in a rat model of type 2 diabetes. Specifically, it seeks to evaluate the cardioprotective and nephroprotective effects of ASTA by examining its impact on renal and cardiac function, oxidative stress markers, and electrocardiographic (ECG) parameters. Additionally, the study aims to elucidate the underlying mechanisms of ASTA’s action, focusing on its effects on antioxidant enzymes namely, NOX4, Fractalkine, Nrf2, and AP-1 involved in diabetic-induced cardiac and renal pathology. Ultimately, this research seeks to provide new insights into the potential of ASTA as a therapeutic approach for managing diabetic complications.

## Material and method

### Animals

The study protocol adhered to the ethical guidelines set forth by the National Research Centre’s (NRC) Ethical Committee in Cairo, Egypt (Approval No. 01480324), and followed the Guidelines for Ethical Conduct in the Care and Use of Non-human Animals in Research of the American Psychological Association. Additionally, this study was conducted and reported in accordance with the ARRIVE guidelines. Adult male Wistar rats (100–120 gm) were procured from the NRC animal house in Cairo, Egypt. Rats underwent a 1-week acclimatization period and were housed under standard conditions (60% relative humidity with a tolerance of ± 10%, temperature maintained at 23 °C with a tolerance of ± 2 °C, and a 12-h light/dark cycle), with ad-libitum access to food and water.

### Chemicals

STZ and ASTA were obtained from Sigma-Aldrich company in in St. Louis, Missouri. Additionally, only molecular or analytical-grade compounds were used.

### Experimental design

#### Induction of diabetic cardiomyopathy

Rats were fed a high-fat/high-fructose (HF/HFr) diet, consisting of a high-fat diet including 14% saturated animal fat, 1% cholesterol powder, 21% protein, 60% carbohydrates, 3% fiber, and 1% vitamins and minerals. In addition, they had ad libitum access to a 20% (w/v) fructose solution for seven weeks. At the beginning of week eight, overnight fasting rats were injected intraperitoneally with a newly prepared single sub-diabetogenic dosage of STZ (35 mg/kg; i.p.) in citrate buffer (pH 4.8, 0.09 M) and given a glucose solution (5% w/w) for the first 24 h^14,15^. The current model reflects human diabetes by combining a high-fat/high-fructose diet to induce insulin resistance, followed by a low-dose STZ to cause mild β-cell dysfunction and stable hyperglycemia, making it suitable for studying disease progression and drug testing (Schaalan, 2009 #1). One week after STZ injection, the T2DM model was verified by testing fasting blood glucose levels with an Accu-Check glucometer (Roche, Mannheim, Germany). Only rats with blood glucose levels ranging from 220 to 300 mg/dl were included in the current study. The animals were afterwards given a regular diet for the remainder of the experiment. Negative control rats feed a standard diet and are given unlimited access to water. At the beginning of the eight^th^ week, these rats given only citrate buffer once (1 ml/kg, i.p.).

#### Study design

**Fig. 1:**
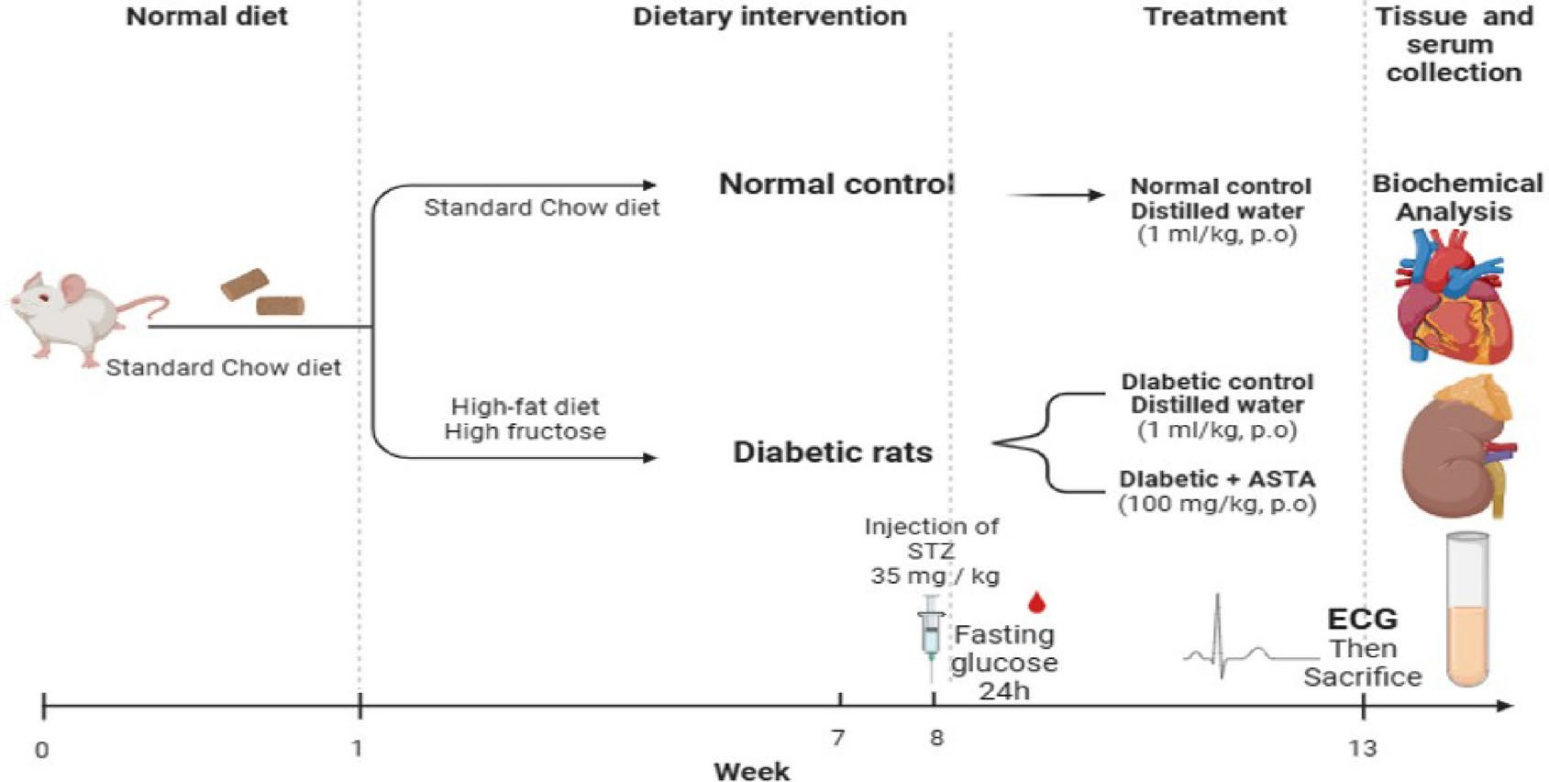
Experimental Design for Induction of Diabetic Cardiomyopathy and Nephropathy and Assessment of Astaxanthin Treatment in Rats. All rats were acclimatized on a standard chow diet during Week 0–1. From Week 1 to 7, diabetic groups were fed a high-fat, high-fructose (HFHF) diet, while the normal control group remained on the standard diet. Diabetes was induced at the end of Week 7 using a single intraperitoneal injection of streptozotocin (STZ; 35 mg/kg), followed by fasting blood glucose measurement after 24 h for inclusion/exclusion criteria. From Week 8 to 13, rats received daily oral treatments: distilled water (1 ml/kg) for control groups and Astaxanthin (100 mg/kg) for the treatment group. Fasting blood glucose was monitored regularly throughout the treatment period. At Week 13, electrocardiography (ECG) was performed before sacrifice, and blood and tissue samples were collected for biochemical, histological, and molecular analyses.

Figure 1 illustrates the experimental timeline, where after the induction of diabetes, the diabetic animals were randomly assigned into three groups: **DC group (Diabetic Control)**: Received distilled water orally (1 ml/kg) and served as the untreated diabetic group. The second group is **ASTA group**: Diabetic rats treated orally with ASTA at a dose of 100 mg/kg. In addition, **control group (Normal Control)**: In which healthy rats being fed a standard diet with free access to water and received distilled water (1 ml/kg) orally. All treatments were administered once daily for four consecutive weeks.

#### **Electrocardiography (ECG)**

Electrocardiography was recorded one day prior to scarification, rats were anesthetized with ketamine (50 mg/kg) and xylazine (5 mg/kg)^16^, and The ECGs were recorded using the PowerLab/8sp system (AD Instruments, Australia), which includes an animal bio-amplifier and LabChart 7 software with an ECG analyzer module which is able to detect the R-R interval, heart rate, QTc, R-amplitude, QRS interval, ST height for rodents and PR interval. Rats were placed in a supine position, and electrodes were attached to the limbs to record standard lead II ECGs.

### Blood and tissue sampling

At the end of the experiment, blood samples were collected from overnight-fasted rats under ketamine/xylazine anesthesia. Blood was withdrawn from the tail vein, and serum was obtained by centrifuging the samples at 3000 rpm for 15 min at 4 °C for biochemical analyses of serum urea, creatinine, Creatine kinase-MB (CK-MB), and lactate dehydrogenase (LDH).

After blood collection, rats were humanely euthanized by cervical dislocation following deep anesthesia with ketamine/xylazine, ensuring minimal distress. The heart, aorta, and kidney tissues were then dissected and washed in saline solution. The parts of isolated organs were kept in 10% formalin for further histological investigation, while the left parts were frozen at −80 °C to assess the following parameters using the homogenate: Malondialdehyde (MDA) level; reduced glutathione (GSH) level, superoxide dismutase (SOD), NOX4, Fractalkine, Nrf2 and AP-1.

### Biochemical analysis

The concentrations of urea and creatinine were determined using commercially available colorimetric assay kits (Biovision, K625-100). The assays were performed according to the manufacturer’s protocols, and absorbance was measured using a microplate reader at the recommended wavelengths. The values were calculated from standard curves generated using known concentrations of urea and creatinine. CK-MB and LDH levels were measured using ELISA kits (Creative Diagnostics, Catalog No. DEIA-FN285) and (FineTest, Calalog No. ER0646). Briefly, serum samples were added to antibody-coated wells and incubated for the binding of CK-MB or LDH. After subsequent washing steps and the addition of enzyme-conjugated secondary antibodies, substrate solution was added to produce a colorimetric reaction.

Tissue levels of oxidative stress and inflammatory markers were determined using ELISA kits according to the manufacturers’ protocols. Malondialdehyde (MDA), was measured calorimetrically (Biovision, Catalog No. K739-100) that utilizes the reaction between MDA and a specific reagent producing a measurable color change; absorbance was read at the designated wavelength and MDA concentrations were calculated from a standard curve. GSH and SOD were quantified via a competitive binding assay (BioVision, Catalog No. K464-100) and (BioVision, Catalog No. K335-100), respectively with optical density measurements used to determine concentrations from a standard curve. Additionally, levels of NOX4 (Novus Biologicals, Catalog No. NBP2-76792) were quantified by measuring optical density and interpolating values from a standard curve. Tissue levels of Fractalkine and Nrf2 were also measured using an ELISA kit (RayBiotech, Catalog No. ELR-Fractalkine) and (Reddot, Catalog No. RD-NFE2L2-Ra), with concentrations determined from a standard curve.

### Western blot analysis of AP-1

Western blotting was performed to assess the expression of AP-1 (c-Jun) in heart and kidney tissues. Tissue homogenates were prepared using RIPA buffer supplemented with protease and phosphatase inhibitors. Protein concentration was determined using the Bradford assay, and equal amounts of protein (e.g., 30–50 µg) were separated by SDS-PAGE and transferred onto PVDF membranes.

Membranes were blocked with 5% BSA in TBST and incubated overnight at 4 °C with a rabbit polyclonal antibody against c-Jun/AP-1 (Proteintech, Catalogue No. 10024-2-AP), which detects both phosphorylated (42–45 kDa) and unphosphorylated (36–39 kDa) forms of c-Jun. The antibody recognizes JUN, a basic leucine zipper (bZIP) transcription factor involved in proliferation, apoptosis, survival, and gene regulation.

Following incubation with an HRP-conjugated secondary antibody, protein bands were visualized using an enhanced chemiluminescence (ECL) detection system. β-actin was used as a loading control. Band intensities were quantified using ImageJ software, and relative protein expression was normalized to β-actin.

### Histopathological examination

All histology samples collected immediately from all rats after sacrifice and fixed for at least 72 h in 10% neutral-buffered formaldehyde. All the specimens were washed for half an hour in tap water and then dehydrated, cleared in xylene and embedded in paraffin. Serial sections of 4 μm thick were cut and stained with hematoxylin and eosin^17^ to examine morphological changes. Images were captured and processed using version8.0 of Adobe Photoshop. The grading study used for assessment of the pathological alterations in cardiac tissue was [–: absence of change, +: 0–30% area shows changes (mild), + +: 30–60% area shows changes (moderate), + + +: 60–100% area shows focal changes (severe focal), + + ++: 60–100% area shows diffuse changes (severe diffuse)]^18^.

Histomorphometry assessment of the kidney was performed under the Leica QWin DW3000 image analysis system (LEICA Imaging Systems Ltd., Cambridge, England) At the pathology lab at the Medical Research Centre of Excellence Unit, National Research Center, by measuring the glomerular diameter, Bowman space, and percentage of proximal tubular necrosis in 5 different X100 fields and the mean of scores per rat was calculated.

Assessment of fibrosis and inflammation in cardiac and renal tissues was performed using a semi-quantitative scoring system. Inflammation was evaluated based on the degree of mononuclear cell infiltration and associated myocyte damage, with scores ranging from 0 to 3. A score of 0 indicated no inflammatory infiltrate, while scores of 1, 2, and 3 corresponded to mild (sparse mononuclear cells without myocyte necrosis), moderate (focal infiltrates with occasional myocyte damage), and severe (diffuse infiltrates with significant myocyte necrosis and disarray) inflammation, respectively. Similarly, fibrosis was assessed by the extent and pattern of collagen deposition in cardiac tissue. A score of 0 indicated no visible fibrosis, whereas mild fibrosis (score 1) was characterized by thin fibrotic strands around blood vessels or scattered interstitial fibrosis. Moderate fibrosis (score 2) involved patchy or multifocal areas affecting larger regions of the myocardium, and severe fibrosis (score 3) was defined by extensive collagen deposition leading to myocardial tissue replacement and pronounced perivascular fibrosis.

### Immunohistochemistry

Sections were incubated with monoclonal antibody against Nrf2 100(Nrf2 Santa Cruiz Laboratory). Sections were then incubated with a biotin conjugated secondary antibody and Streptavidin Enzyme Conjugate. The reaction sites were seen as nuclear brown staining. Immunohistochemical staining of cardiac and renal tissues for Nrf2 was evaluated semi-quantitatively in a blinded manner using a 0–4 scoring system, based on the percentage of positively stained cells: 0 for 0–10%, 1 for 11–25%, 2 for 26–50%, 3 for 51–75%, and 4 for > 75% staining^19^.

### Statistical analysis

All of the values were presented as Mean ± SD. Data was tested for normality using the Shapiro-Wilk test to ensure the data followed a normal distribution. Graph Pad Prism (version 9.0; San Diego, CA, USA) was used for the statistics analysis. For multiple comparisons, the Analysis of Variance (ANOVA) test was used, after that, the Tukey-Kramer test was used as a post-ANOVA test. A p-value less than 0.05 was considered statistically significant. For the quantitative histopathological analysis, statistical evaluation was conducted using the Kruskal-Wallis test for non-parametric data and one-way ANOVA for parametric data, followed by appropriate post hoc multiple comparison tests. A p-value less than 0.0001 was considered statistically significant.

## Results

### Effect of ASTA on cardiac rhythmicity and contractility of hf/hfr/stz induced diabetic complications in rats

**Fig. 2:**
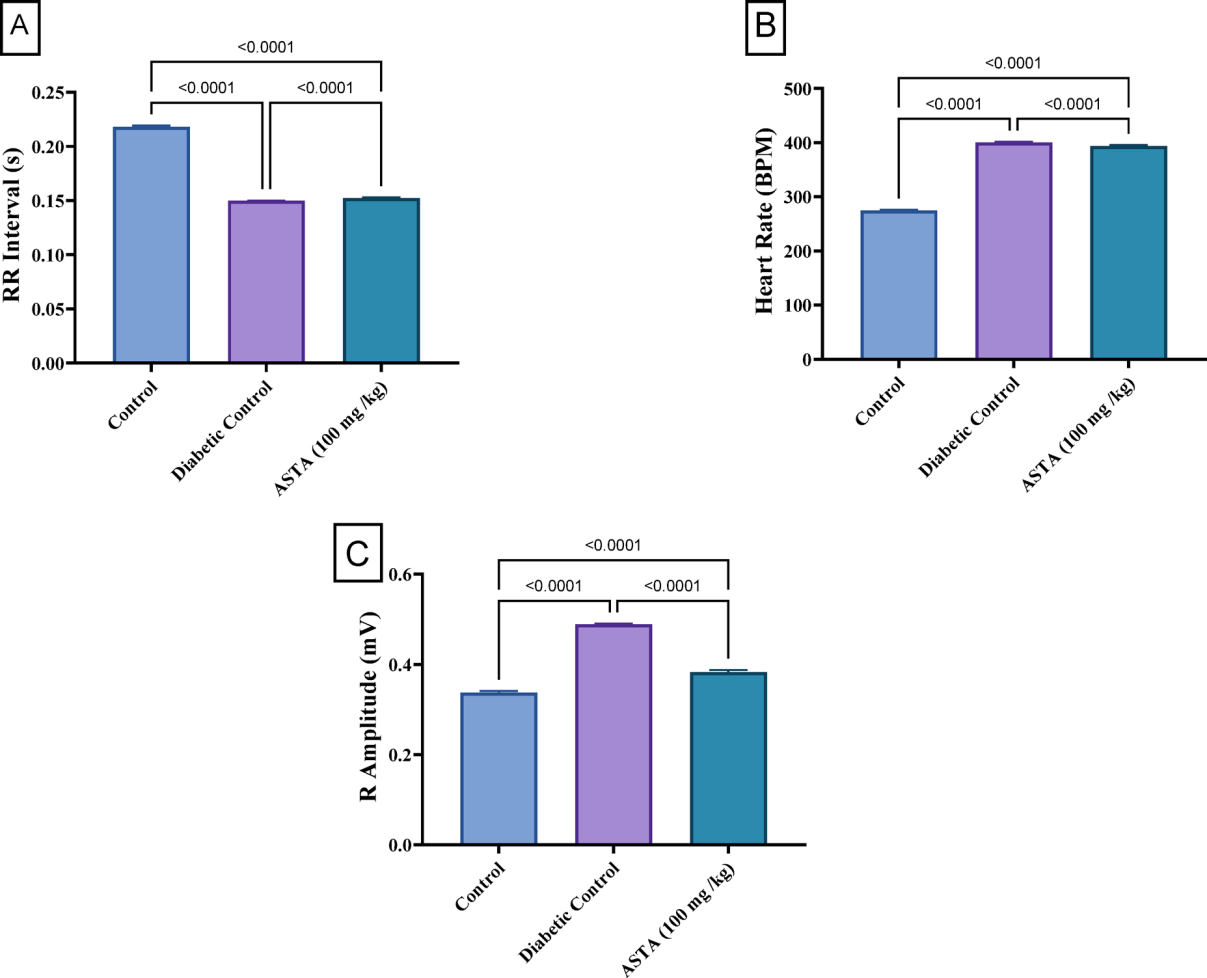
Effect of ASTA on Cardiac Rhythmicity and Contractility of HF/HFr/STZ induced Diabetic Complications in Rats. (**A**) RR interval; (**B**) Heart Rate and (**C**) R Amplitude. Data are presented as mean ± standard deviation (SD). Statistical analysis was performed using One-way analysis of variance (ANOVA) followed by Tukey-Kramer post hoc test was used for multiple comparisons. Differences were considered statistically significant at *P* < 0.05. *Significant difference compared to control groups.

As illustrated in figure 2, the DC group demonstrated significant alterations in cardiac rhythmicity and contractility compared to the normal group. Hence, the RR interval; a key measure of cardiac rhythmicity, indicating the time between successive heartbeats, reflecting autonomic regulation, decreased by 31.3%, and resulted in a 45.7% increase in heart rate; which is a critical indicator of cardiac rhythm and function, representing the number of heart beats per minute. These results confirm a disruption in cardiac autonomic regulation in diabetic group.

Furthermore, the R amplitude, which is vital for efficient cardiac output. It reflects the myocardial contractility and the strength of the heart’s contraction. R amplitude increased nearly by 44.7% in the DC rats. On the other hand, treatment with ASTA (100 mg/kg) partially normalizing rhythmicity and reversed these changes. It increased the RR interval by 12% and decreased the heart rate by 10.7% compared to DC, although these parameters remained significantly different from normal indicating that has minimal effects on RR interval and heart rate. Regarding contractility, ASTA was able to reduce the R amplitude compared to DC by 21.7%.

### Effect of ASTA on cardiac conduction and repolarization of hf/hfr/stz induced diabetic complications in rats

**Fig. 3:**
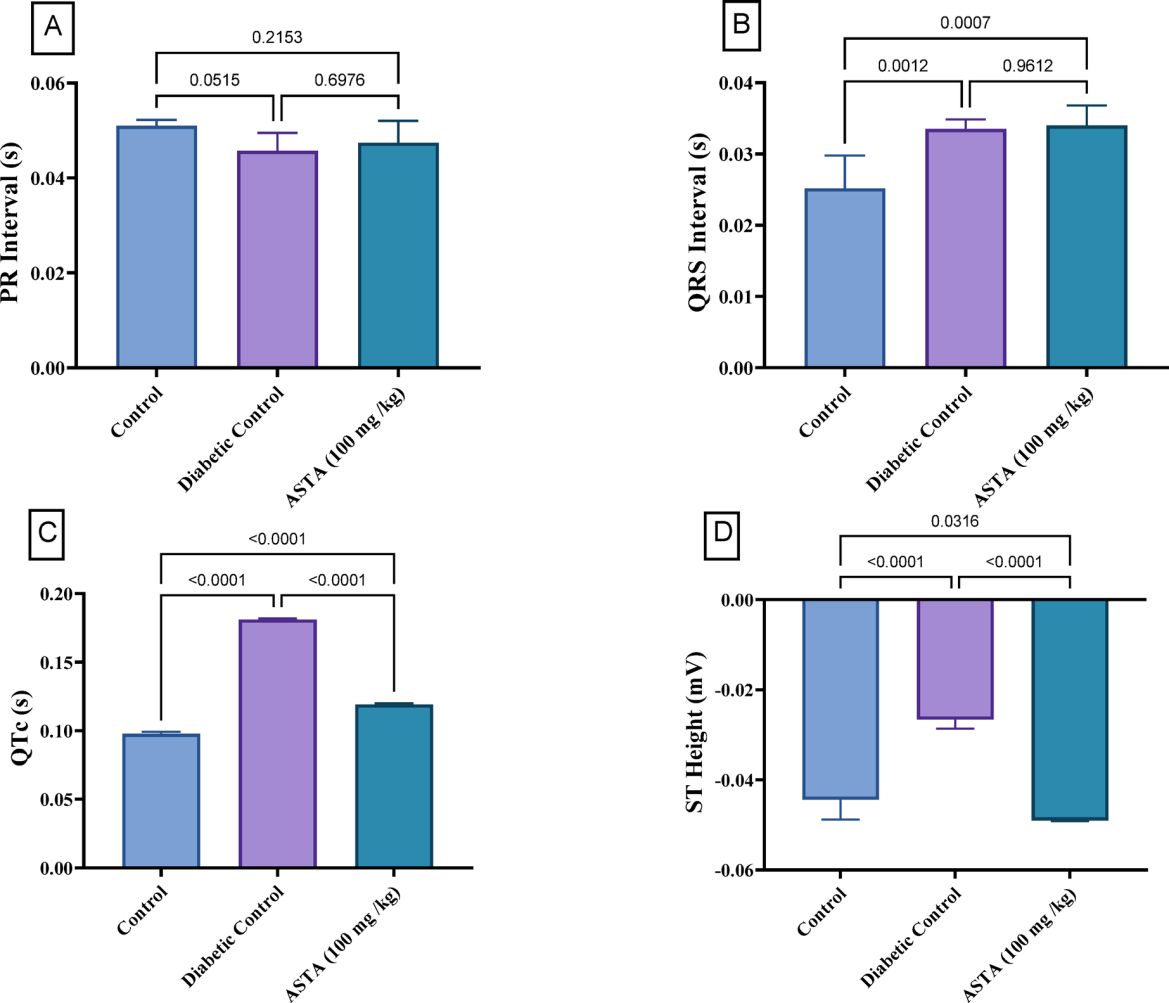
Effect of ASTA on Cardiac Conduction and Repolarization of HF/HFr/STZ induced Diabetic Complications in Rats. (**A**) PR interval; (**B**) QRS Interval (**C**) QTc and (**D**) ST Height. Data are presented as mean ± SD. Data are presented as mean ± standard deviation (SD). Statistical analysis was performed using One-way analysis of variance (ANOVA) followed by Tukey-Kramer post hoc test was used for multiple comparisons. Differences were considered statistically significant at *P* < 0.05. *Significant difference compared to control groups.

Figure 3 presents the HF/HFr/STZ induced DCM in rats exhibited significant changes in cardiac conduction and repolarization. The PR interval decreased by 10.4%, QRS interval increased by 32.9%, QTc increased by 85.1%, and ST height became less negative by 40.1% compared to normal. However, administration of ASTA (100 mg/kg) had varying effects on these parameters. The PR interval was further reduced, while the QRS interval was minimally affected by ASTA treatment. Notably, the QTc interval was significantly improved with ASTA, showing a 34.2% reduction. However, QTc remained elevated compared to normal. Additionally, the ST height became more negative with ASTA treatment, displaying an 84.2% change. Overall, while ASTA improved certain aspects of cardiac conduction and repolarization, it did not fully normalize these parameters to control levels.

### Effect of ASTA on blood glucose level and serum markers of cardiac and renal function of hf/hfr/stz induced diabetic complications in rats

**Fig. 4:**
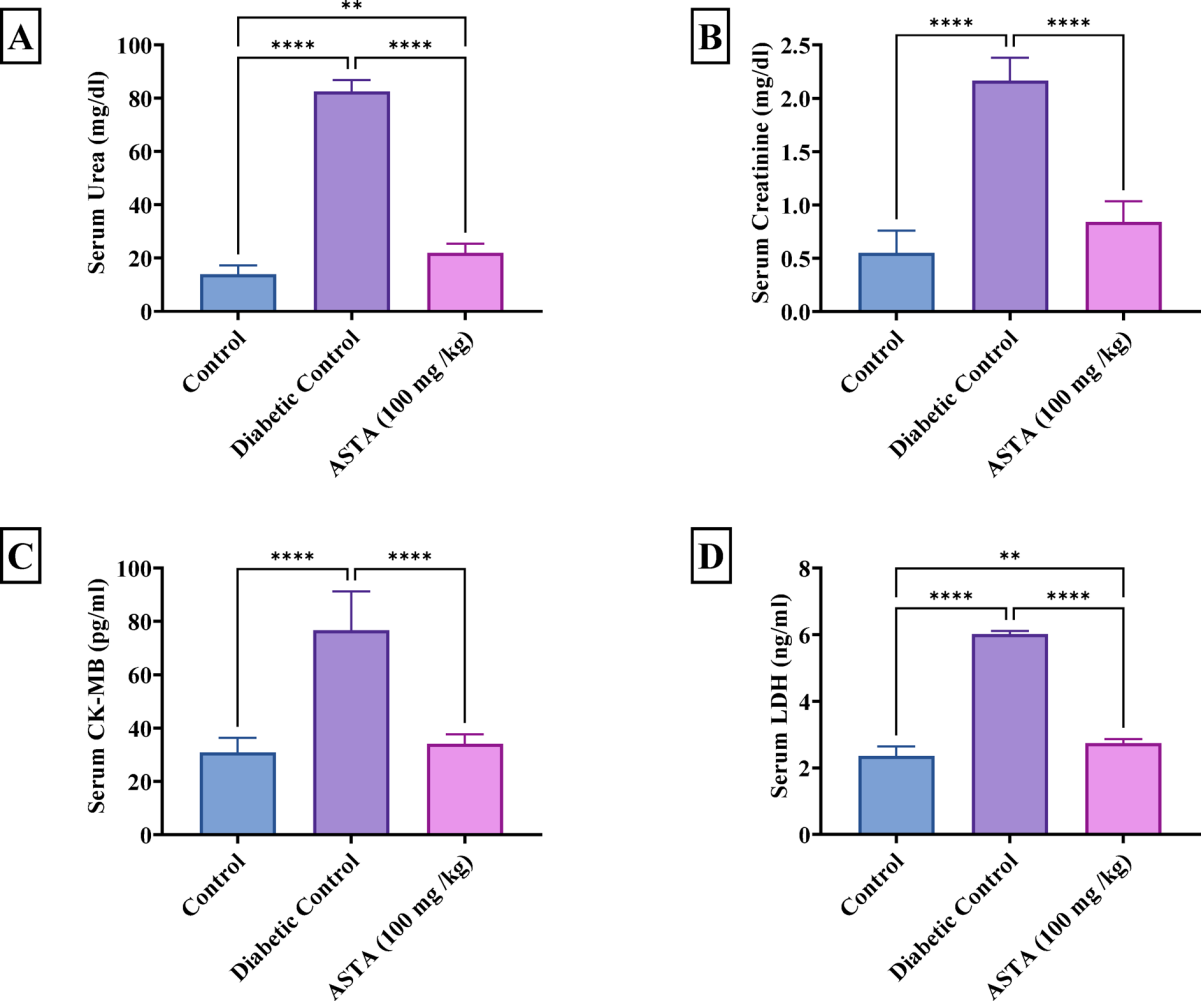
Effect of ASTA on Serum Markers of Cardiac and Renal Function of HF/HFr/STZ induced Diabetic Complications in Rats. Serum urea (**A**) Serum Creatinine (**B**); Cardiac creatine phosphokinase-MB (CK-MB) (**C**); Lactate dehydrogenase (LDH) (**D**). Data are presented as mean ± standard deviation (SD). Statistical analysis was performed using One-way analysis of variance (ANOVA) followed by Tukey-Kramer post hoc test was used for multiple comparisons. Differences were considered statistically significant at *P* < 0.05. *Significant difference compared to control groups.

Blood glucose levels confirm the model, with the DC group exhibiting significantly higher levels (263.82 ± 8.64 mg/dl) compared to the normal control group (98.14 ± 1.76 mg/dl), while ASTA treatment reduced glucose levels to 187.85 ± 14.38 mg/dl. These findings collectively indicate that ASTA not only provides protective effects on myocardial and renal functions but also significantly improves glucose metabolism in diabetic rats **(**Table 1**)**. As depicted in figure 4 the DC group showed significant increases in serum CK-MB; serving as a marker for cardiac muscle damage, LDH, urea, and creatinine approximately by 2-, 2-, 5-and 3-fold, respectively suggested tissue damage and indicated kidney impairment beside reduced glomerular filtration rate as compared to the normal control group. In contrast, treatment with ASTA diminished the elevated CK-MB by 56%, LDH by 54%, serum urea by 73%, and creatinine by 61%, compared to DC, bringing levels close to normal control values, indicating a protective effect on both myocardial and renal functions.

**Table 1:** Effect of ASTA on blood glucose level of hf/hfr/stz induced diabetic complications in Rats.

Groups	Blood glucose level (mg/dl)
Control	98.14 ± 1.76
Diabetic Control	263.82 ± 8.64
ASTA (100 mg/kg)	187.85 ± 14.38

### Effect of ASTA on oxidative stress markers in heart and kidney of hf/hfr/stz induced diabetic complications in rats

Induction of diabetic complications (DCM and DN) in rats was exhibited markedly increased MDA levels (nmol/mg protein) in heart and kidney by 6 and 5-fold, respectively. MDA is a marker of lipid peroxidation, and its elevated levels reflect cellular damage due to free radicals and reactive oxygen species. This was accompanied by a concurrent decrease in GSH (nmol/mg protein) levels in both organs, by 76% and 77%, respectively, reflecting a reduction in the body’s antioxidant defense. GSH is a crucial antioxidant that protects cells from oxidative damage, and its decreased levels indicate diminished antioxidant defenses and triggers the oxidative stress.

**Fig. 5:**
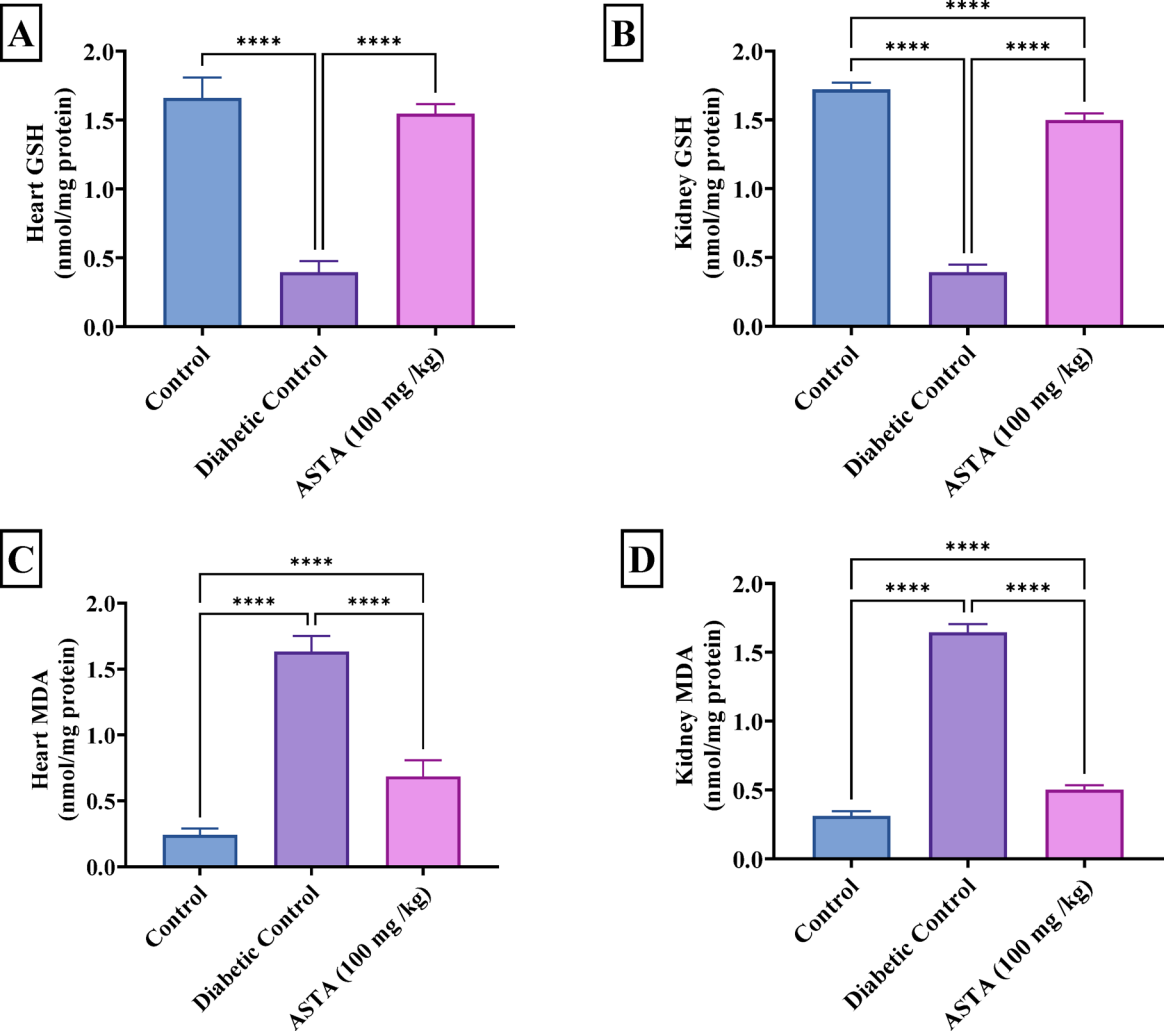
Effect of ASTA on Oxidative Stress Markers in Heart and Kidney of HF/HFr/STZ induced Diabetic complications in Rats. Cardiac GSH (**A**); Renal GSH (**B**); Cardiac MDA (**C**); Renal MDA (**D**). Data are presented as mean ± standard deviation (SD). Statistical analysis was performed using One-way analysis of variance (ANOVA) followed by Tukey-Kramer post hoc test was used for multiple comparisons. Differences were considered statistically significant at *P* < 0.05. *Significant difference compared to control groups.

Treatment with ASTA (100 mg/kg) significantly resulted in reducing the heart and kidney MDA content by 58% and 70%, respectively while increasing both the heart and kidney GSH content almost by 3-fold as compared to DC besides brought levels close to normal as shown in figure 5. These results suggest that ASTA may help alleviate oxidative stress and restore the balance between pro-oxidants and antioxidants in both the heart and kidney.

### Effect of ASTA on antioxidant enzymes and NOX4 in heart and kidney of hf/hfr/stz induced diabetic complications in rats

**Fig. 6:**
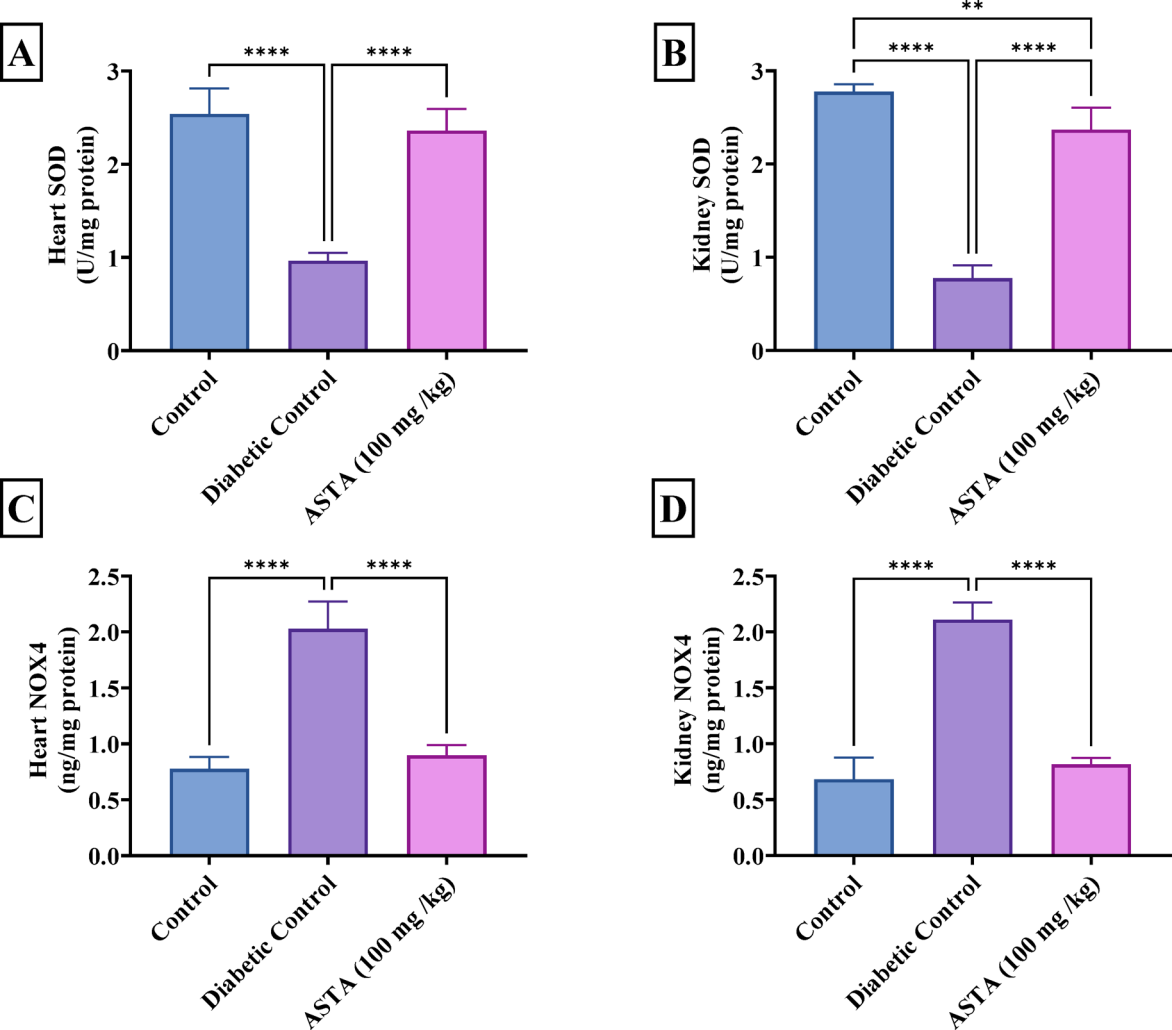
Effect of ASTA on Antioxidant Enzymes and NOX4 in Heart and Kidney of HF/HFr/STZ induced Diabetic Complications in Rats. Cardiac SOD (**A**); Renal SOD (**B**); Cardiac Nox4 (**C**); Renal NOX4 (**D**). Data are presented as mean ± standard deviation (SD). Statistical analysis was performed using One-way analysis of variance (ANOVA) followed by Tukey-Kramer post hoc test was used for multiple comparisons. Differences were considered statistically significant at *P* < 0.05. *Significant difference compared to control groups.

Figure 6 illustrates the impact of diabetic complications induced by HF/HFr/STZ on antioxidant enzymes and NOX4 levels, revealing a significant reduction in SOD activity (µ/mg of protein) in both the heart and kidney in DC rats, nearly by 62% and 72%, respectively. SOD is a key antioxidant enzyme that catalyzes the conversion of superoxide radicals to hydrogen peroxide, and its decreased activity indicates an impaired ability to neutralize oxidative stress. Additionally, NOX4 levels increased by 2- and 3-fold in the heart and kidney, respectively, compared to the normal control. NOX4 is an enzyme that contributes to the production of reactive oxygen species (ROS), and its elevated levels indicate an increase in oxidative stress and inflammation. In contrast, treatment with ASTA reversed these changes by increasing heart and kidney SOD levels by 2- and 3-fold, respectively, indicating enhanced antioxidant capacity. Furthermore, ASTA decreased NOX4 content in the heart and kidney by 56% and 61%, respectively, compared to the DC group, suggesting a reduction in oxidative stress and inflammation. These results indicate that ASTA has a protective effect by enhancing antioxidant defenses and reducing ROS production in both the heart and kidney.

### Effect of ASTA on chemokine fractalkine levels in heart and kidney of hf/hfr/stz induced diabetic complications in rats

**Fig. 7:**
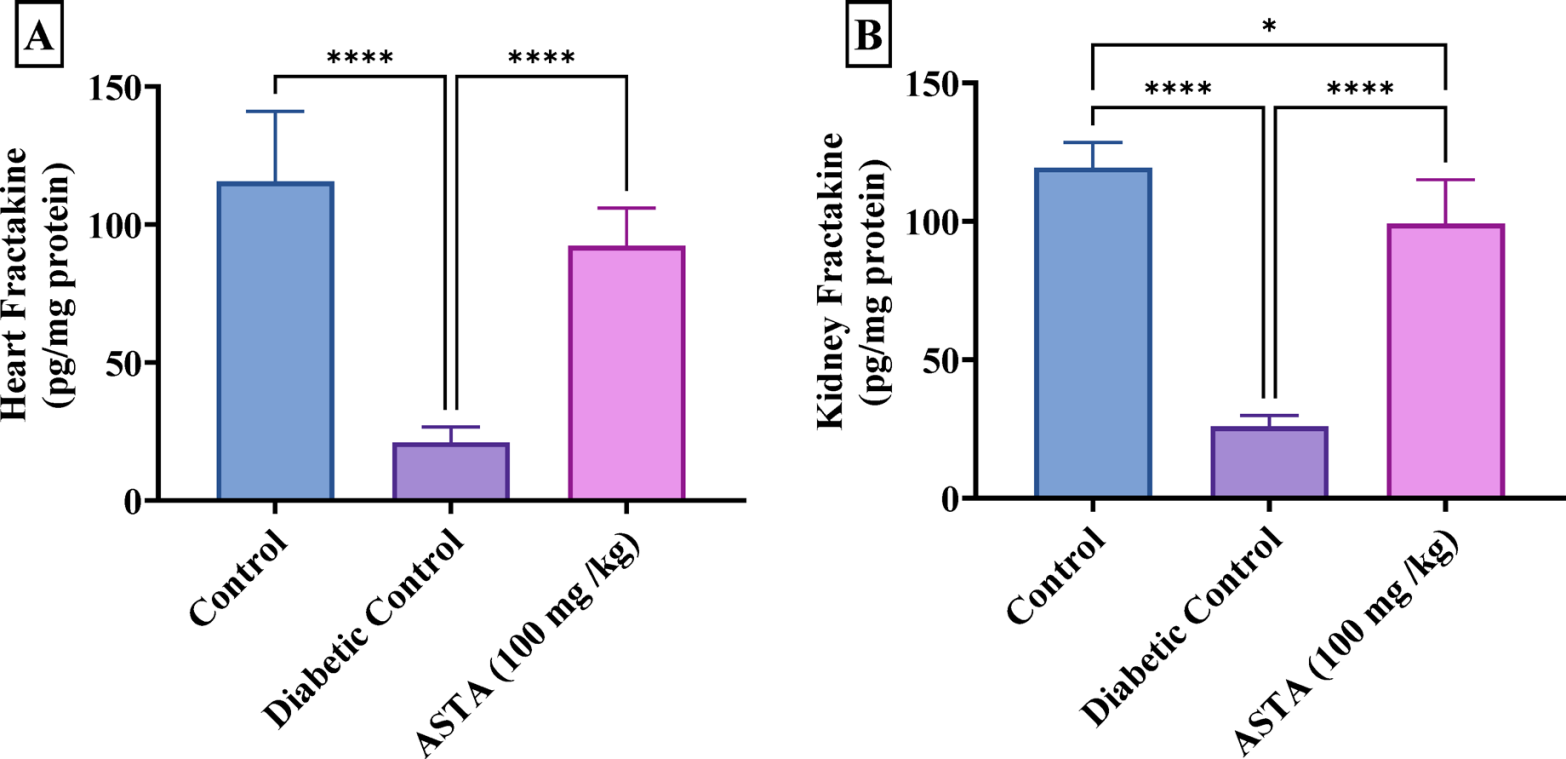
Effect of ASTA on Chemokine Fractalkine Levels in Heart and Kidney of HF/HFr/STZ induced Diabetic Complications in Rats. Cardiac Fractalkine (**A**); Renal Fractalkine (**B**). Data are presented as mean ± standard deviation (SD). Statistical analysis was performed using One-way analysis of variance (ANOVA) followed by Tukey-Kramer post hoc test was used for multiple comparisons. Differences were considered statistically significant at *P* < 0.05. *Significant difference compared to control groups.

Diabetic control rats showed dramatic decreases in the chemokine Fractalkine levels in both the heart and kidney, by 82% and 78%, respectively, compared to the normal control. Fractalkine is a chemokine involved in the recruitment and activation of immune cells, particularly in inflammation and tissue repair. A decrease in its levels suggests an impaired immune and inflammatory response as well as reduced tissue repair capacity. Treatment with ASTA significantly elevated Fractalkine levels in the heart by 4-fold and in the kidney by 3-fold compared to the DC group, bringing these levels closer to normal, as demonstrated in Fig. 7. This suggests that ASTA may help restore immune function and promote tissue repair in both the heart and kidney under diabetic complications.

### Effect of ASTA on transcription factors Nrf2 and AP-1 in heart and kidney of hf/hfr/stz induced diabetic complications in rats

**Fig. 8:**
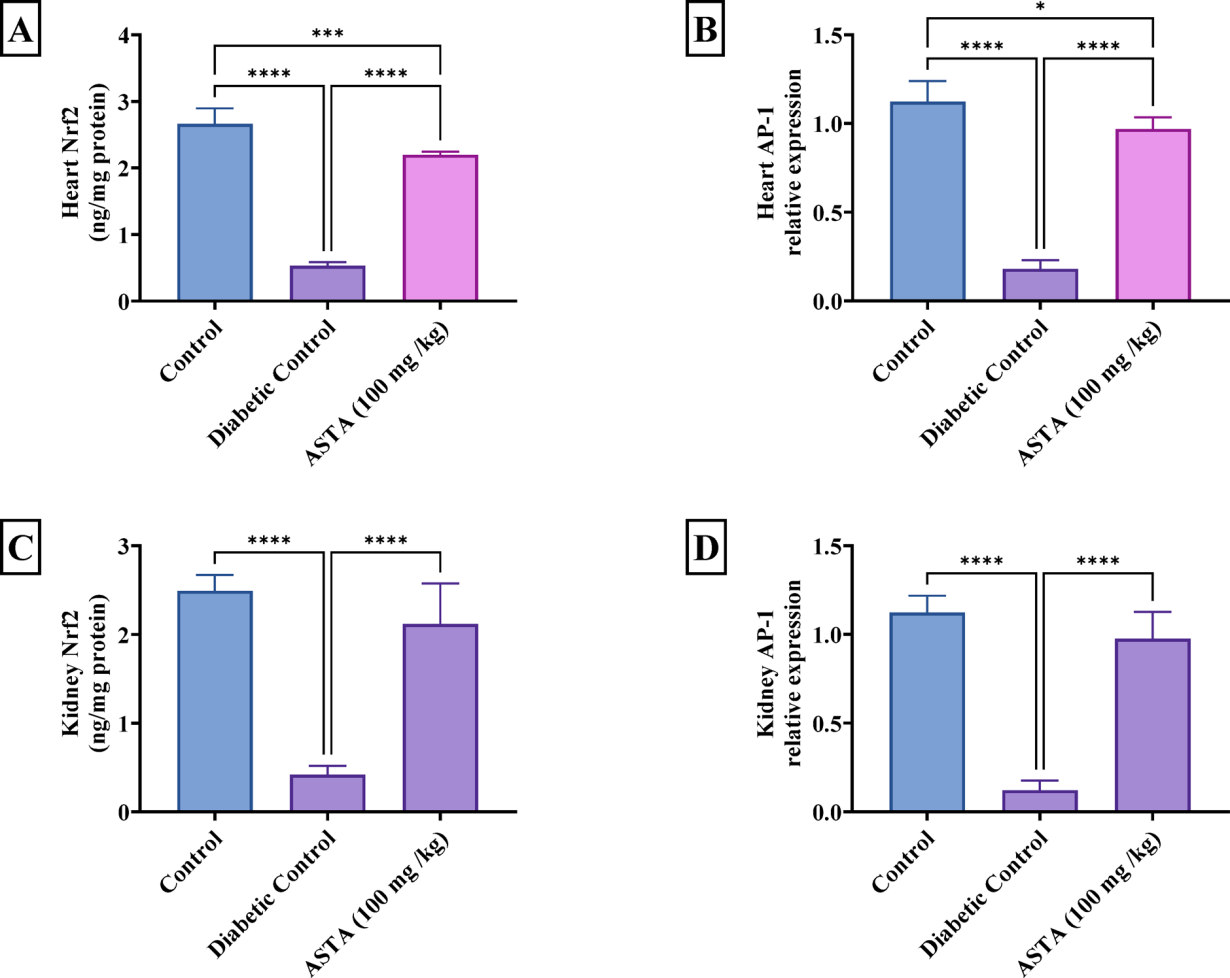
Effect of ASTA on Transcription Factors Nrf2 and AP-1 in Heart and Kidney of HF/HFr/STZ induced Diabetic Complications in Rats. Cardiac Nrf2 (**A**); Renal Nrf2 (**B**); Cardiac AP-1 (**C**); Renal AP-1 (**D**). Data are presented as mean ± standard deviation (SD). Statistical analysis was performed using One-way analysis of variance (ANOVA) followed by Tukey-Kramer post hoc test was used for multiple comparisons. Differences were considered statistically significant at *P* < 0.05. *Significant difference compared to control groups.

Diabetic rats displayed (Fig. 8) significant decrease in Nrf2 and relative protein expression of AP-1 levels in both heart (80% and 84% reductions, respectively) and kidney (83% and 89% reductions, respectively) compared to normal control. Nrf2 is a key transcription factor involved in the activation of antioxidant response elements, regulating the body’s defense against oxidative stress, while AP-1 is a transcription factor that plays a critical role in regulating inflammatory responses and cell proliferation, its expression was analyzed using Western blotting. The reduction in these transcription factors indicates a compromised cellular defense and turn-on the inflammatory cascades.

On other hand, administration of ASTA markedly increased these transcription factors proven by increasing both heart Nrf2 and AP-1 by 4- and 5-fold, respectively, besides elevating the kidney Nrf2 and AP-1 content by 5- and 8-fold, respectively, as compared to DC nearly to normal control suggesting that ASTA was able to restore the activity of key transcription factors involved in oxidative stress defense and inflammation, thereby promoting better cellular protection against the diabetic complications.

### Histopathological and immunohistochemical analysis of heart and kidney on hf/hfr/stz induced diabetic complications in rats

**Fig. 9:**
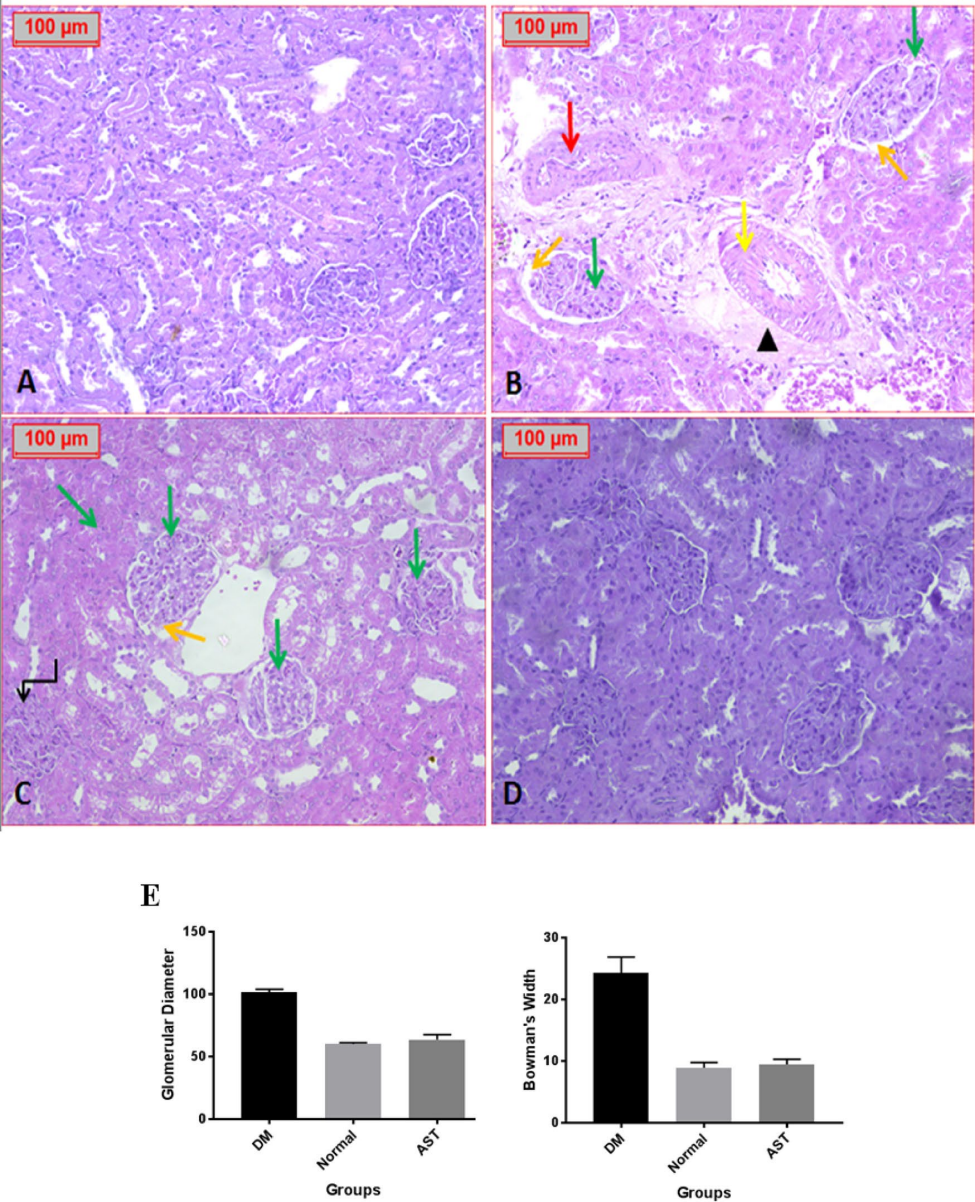
Effect of ASTA on Heart and Kidney histopathological alterations of HF/HFr/STZ induced Diabetic Complications in Rats. photomicrographs show (**A**): Normal control group in which have normal renal parenchyma. (**B** & **C**): Positive diabetic control group which showed increased capillary thickening (red arrow), fibrosis (arrowhead), increase in tubular thickening (yellow arrow), Boman’s space (orange arrow), inflammatory, glomerulosclerosis (green arrow) and glomeruli with lost capsule (broken arrow). (**B** & **C**): Treated group with ASTA showed normal renal parenchyma (**D**). (**H** & **E**, x100).

**Fig. 10:**
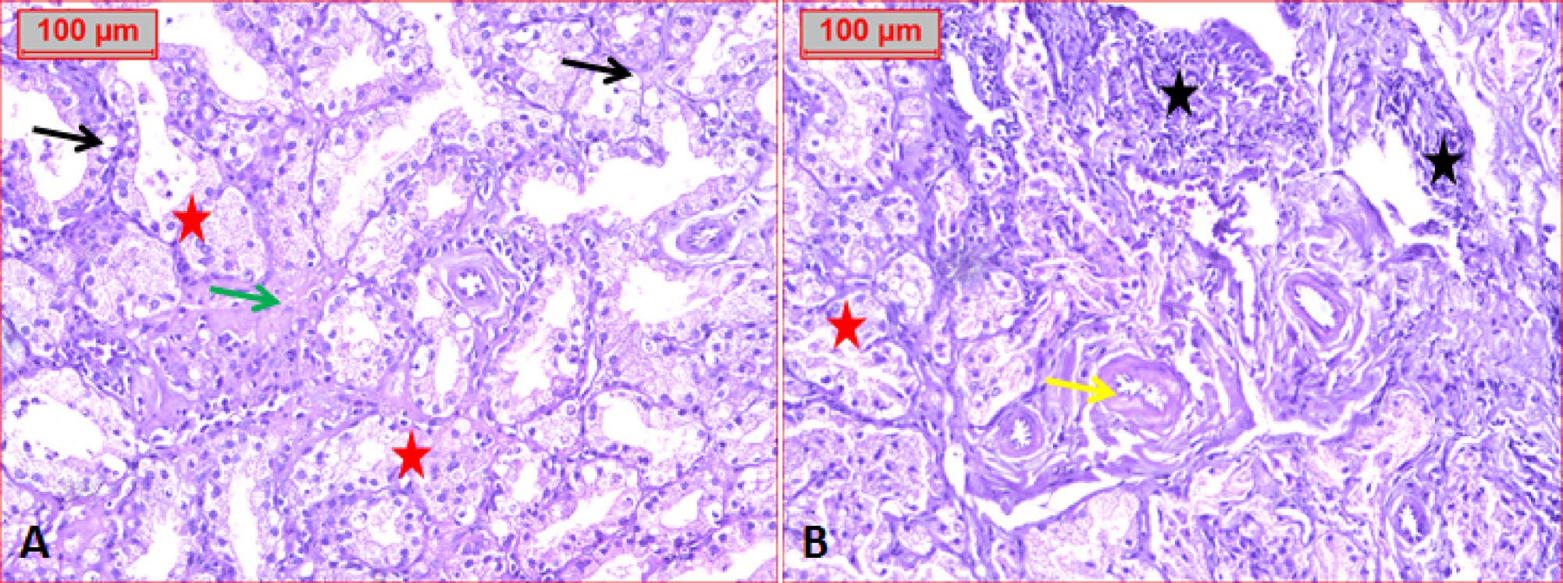
Photomicrographs showed: Positive diabetic group in which tubular destruction with intratubular hyaline cast (red star). In Addition, amyloid deposition (green arrow), inflammatory cell infiltrate (black star), scattered vacuoles and thickened capillary wall (yellow arrow) were noticed (H&E, x100).

Histopathological evaluation of renal tissue in the normal control group revealed normal renal parenchyma (Fig. 9A). In the diabetic group, significant structural changes were observed, including an increase in glomerular diameter and Bowman’s space width, with mesangial expansion, proliferation, and glomerulosclerosis. Some glomeruli exhibited capsule loss, and there was increased glomerular capillary thickening, tubular thickening, and fibrosis (Fig. 9B & C). These changes are indicative of diabetic nephropathy. However, treatment with ASTA resulted in marked improvement of the renal parenchyma, with significant reductions in glomerular diameter (P˂0.0001) and Bowman’s space width (P˂0.0001) (Fig. 9D & E), bringing them closer to normal levels. In the diabetic group, proximal tubular destruction was evident, with the presence of intratubular hyaline casts, inflammatory cell infiltration, and moderate scattered vacuoles (Fig. 10), reflecting renal damage. Treatment with ASTA significantly mitigated these pathological changes, suggesting a protective effect on kidney structure.

**Fig. 11:**
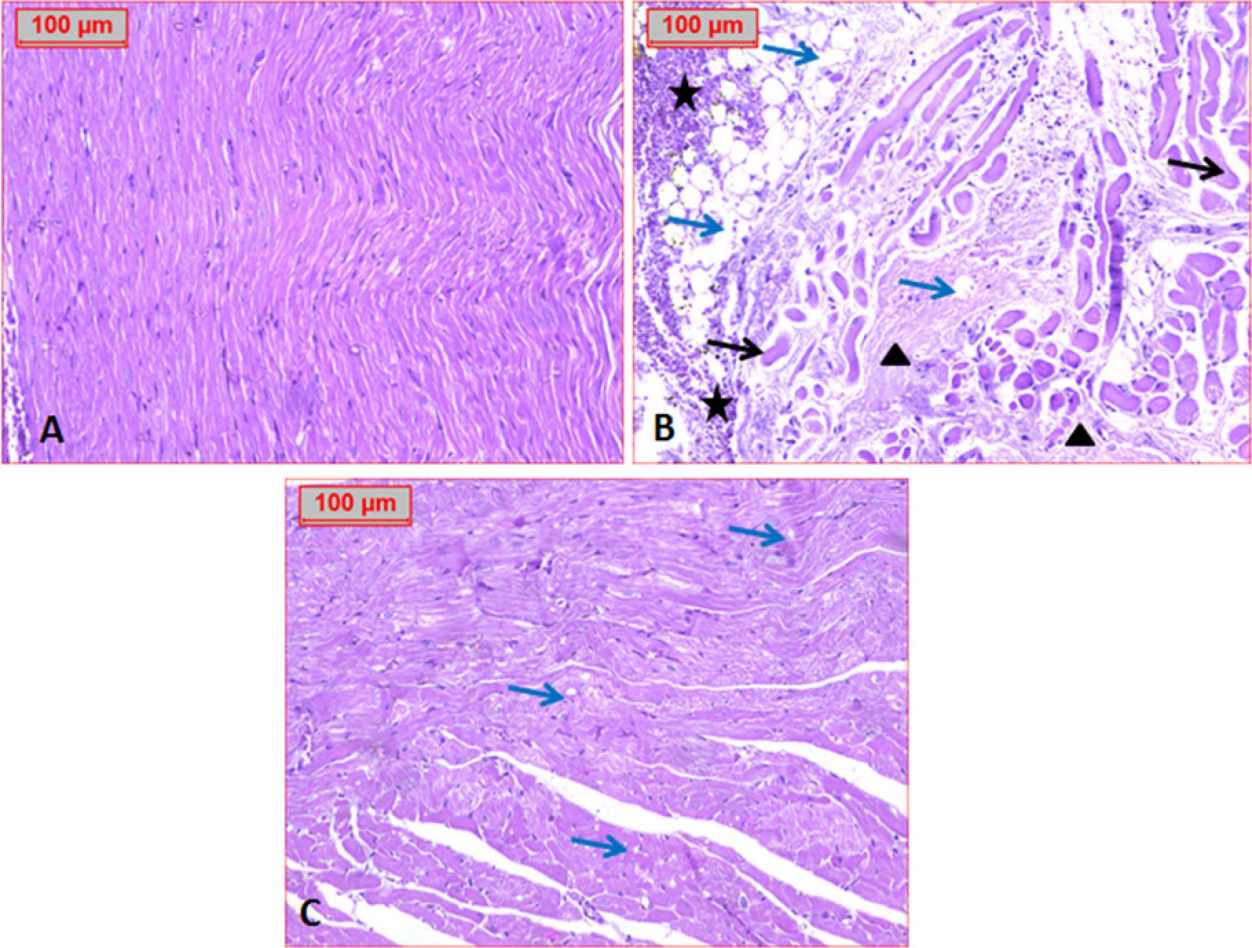
Photomicrograph illustrated: (**A**) Normal control group. (**B**) Positive control group which showed cytoplasmic vacuolization, fibrosis, myocardial cell necrosis, inflammatory cell infiltrate. (**C**) Treated group with ASTA demonstrated scattered vacuoles and inflammatory cells (H&E, x100). Blue arrow: vacuolization, arrowhead: and star: inflammatory cell infiltrate.

Histopathological analysis of cardiac tissue from the normal control group showed normal heart tissue structure without inflammation (graded as -: absence of changes) (Fig. 11A). In the diabetic group, structural abnormalities were observed, including necrosis, signs of myocardial cell death, nuclear lysis, scattered cells with cytoplasmic vacuolization, fibrosis, and myocardial inflammation (++), indicating that 30–60% of the heart tissue was damaged, confirming cardiotoxicity (Figure 11B). In contrast, the ASTA-treated group exhibited significant improvement in cardiac tissue, with mild scattered vacuolization and mild inflammation (+), indicating that inflammation was reduced to 0–30% (Figure 11C). These findings suggest that ASTA has a protective effect on the heart, reducing inflammation and tissue damage in diabetic complications.

**Fig. 12:**
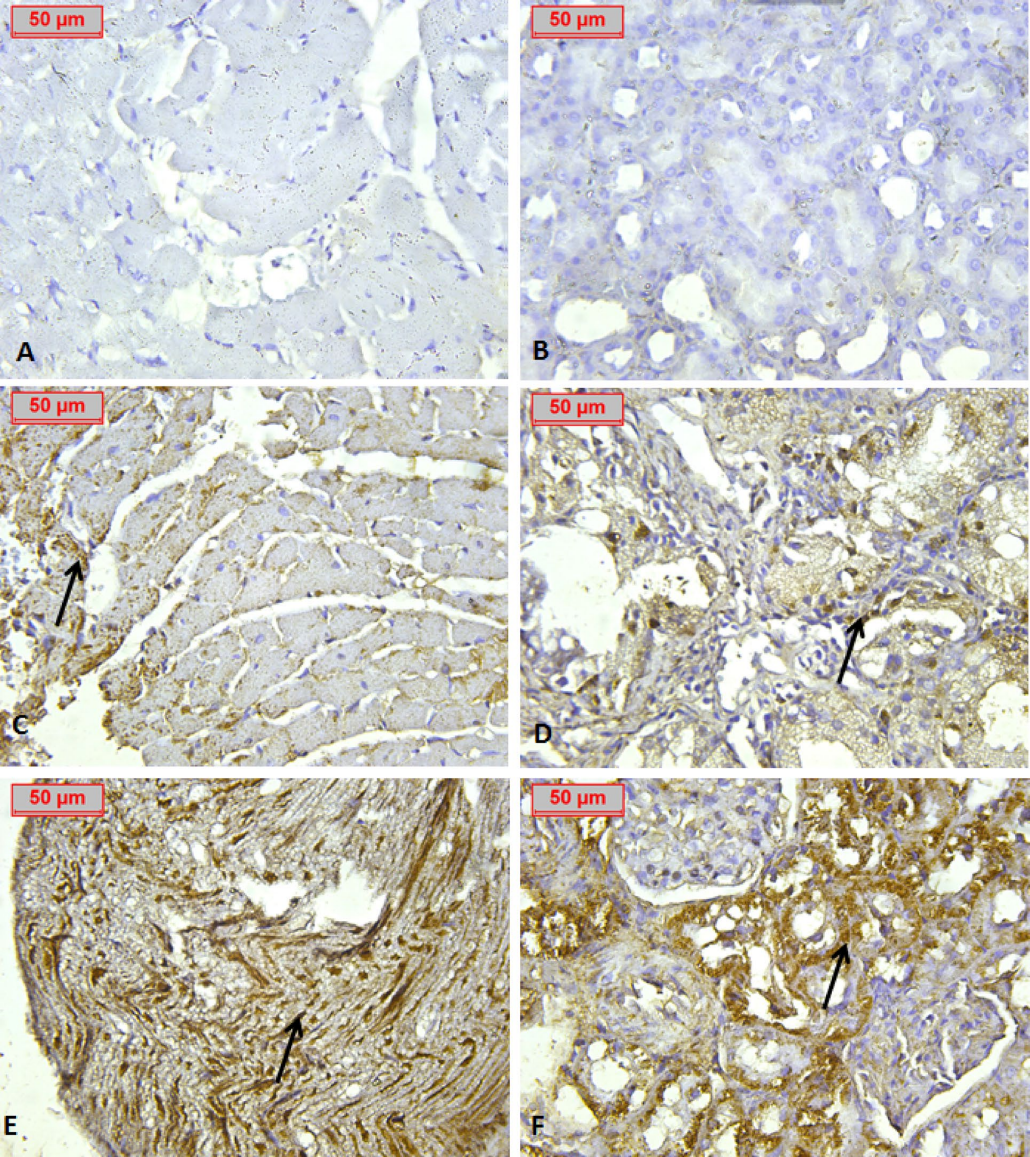

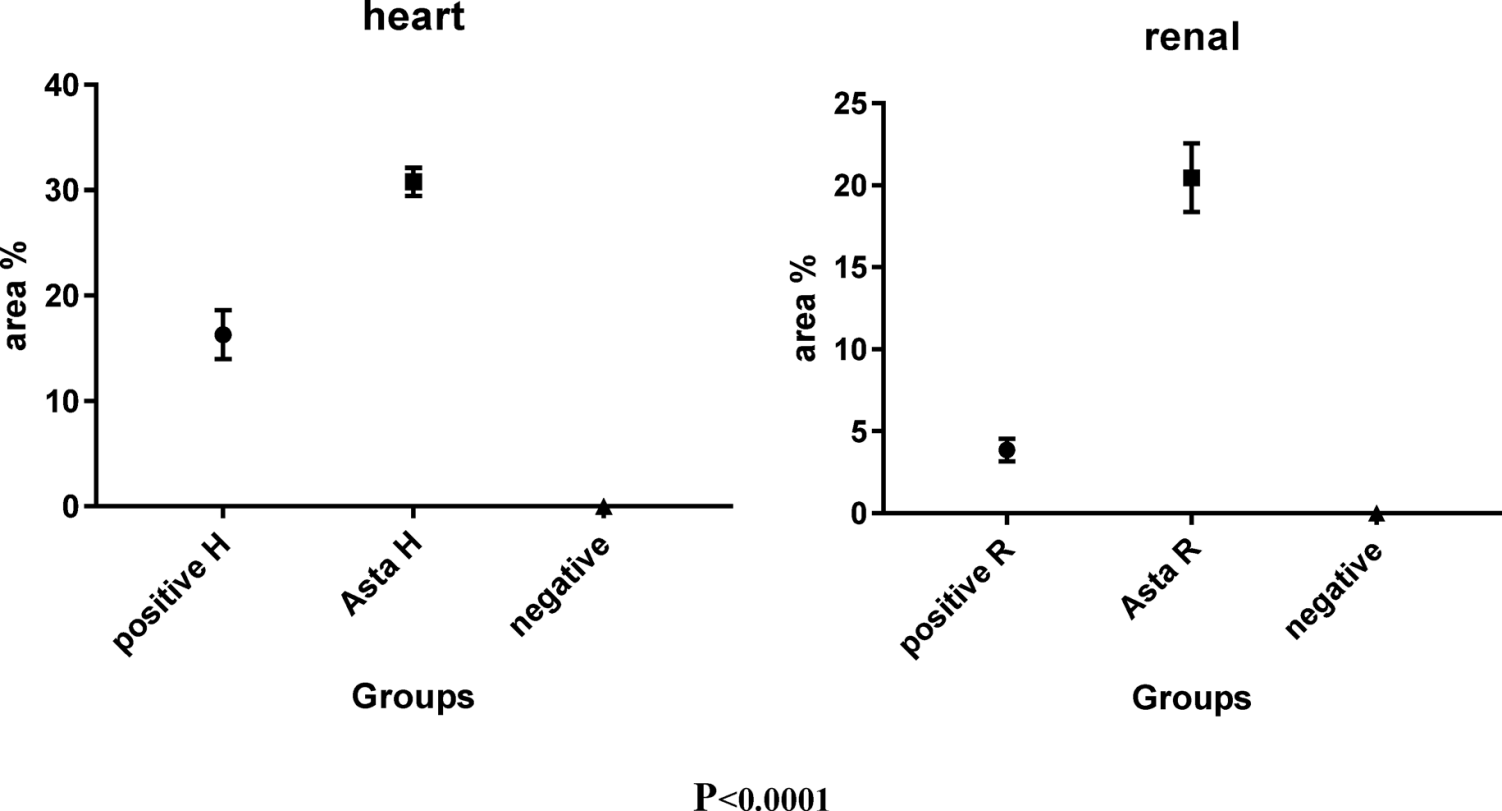
Photomicrograph of Immunohistochemical study of Nrf2 in different groups of cardiac and renal tissues which revealed the following: (**A**, **B**): Negative groups showed negative expression. (**C**, **D**): Positive groups revealed mild expression. (**E**, **F**): Showed high expression in the cardiac tissue and moderate tissue expression in renal tissue. Arrows shows Nrf2 nuclear immunoreactivity (IHC Nrf2, x400).

Immunohistochemical analysis was conducted to assess Nrf2 expression in both heart and kidney tissues. Normal control groups for both organs displayed no significant Nrf2 expression (Fig. 12A, B). In contrast, the positive control groups exhibited mild Nrf2 positivity, with a notably higher expression in the cardiac tissue compared to the renal tissue (Fig. 12C, D). Furthermore, groups treated with ASTA demonstrated enhanced immunoreactivity, with a stronger presence of Nrf2 in the cardiac tissue relative to the renal tissue (Fig. 12E, F).

## Discussion

This study represents the efficacy of ASTA in the mitigation of diabetic cardiomyopathy and diabetic nephropathy using a rat model of type 2 DM. ASTA treatment improved cardiac and renal function, markers of oxidative stress, and histopathological changes, indicating this compound had a multidimensional protective effect against diabetes-induced complications. The findings of this study demonstrate the therapeutic potential of ASTA in mitigating DCM and DN in a rat model of T2DM. ASTA treatment effectively improved multiple key indicators of cardiac and renal function. Moreover, ASTA administration showed substantial reductions in oxidative stress markers and inflammatory cytokines, both critical contributors to DM-related organ damage. Additionally, ASTA improved cardiac electrophysiological stability and renal filtration markers, indicating a protective effect on tissue integrity and function at a dose 100 mg/kg. This dose was selected based on previous preclinical reports demonstrating its efficacy and safety in experimental models of diabetes-related oxidative damage and inflammation. Although this dose is considered high, it is often employed in rodent studies to compensate for ASTA’s relatively low oral bioavailability, which limits its systemic availability and therapeutic concentrations in target tissues^20^. These results offer promising insights into ASTA’s potential as a therapeutic intervention for DM-induced complications, highlighting its role in addressing both structural and functional damage associated with chronic hyperglycemia. DMC and DN are driven by a combination of hyperglycemia-induced oxidative stress, inflammation, and dysregulated cellular signaling, all leading to myocardial^12^ and renal dysfunction^21^.

In this study, DC rats exhibited significant impairments in cardiac rhythmicity and contractility, as evidenced by alterations in ECG parameters such as the RR interval, QTc, and ST height. These results are consistent with the electrophysiological disturbances commonly observed in DC, including prolonged QT intervals and altered repolarization patterns^22–24^, which increase the risk of arrhythmias and sudden cardiac death^25^. On the other hand, ASTA demonstrated significant cardioprotective effects in diabetic rats, notably improving cardiac contractility and rhythmicity. The ASTA emerged as effective in enhancing the RR interval and reducing heart rate, highlighting its potential as a therapeutic agent for diabetes-induced cardiac dysfunction. Although these parameters did not completely normalize, the pronounced improvements underscore ASTA’s ability to partially reverse the deleterious effects of diabetes on cardiac function. The findings align well with prior studies documenting the cardioprotective effects of ASTA across various models of cardiovascular dysfunction. For instance, Zhang and Xu (2018) reported that ASTA improved heart rate variability and mitigated myocardial injury in diabetic rats, largely by reducing oxidative stress and inflammation—two critical contributors to diabetic cardiomyopathy^26^. Similarly, it has been demonstrated that ASTA enhancing cardiac function and reducing myocardial infarct size, further supporting its broad cardioprotective properties^27^. It has also been suggested that ASTA significantly blood pressure reduction and showed a delayed incidence of stroke^28^.

In contrast, a previous study has used ASTA at a dose of 100 mg/kg and reported that ASTA reduces type 2 DM‑associated cognitive decline in rats^29^. Furthermore, it may exert in the same dose neuroprotective effects and improve cognitive function^30^. Similarly, Kumar et al. found that oral administration of ASTA (100 mg/kg) exhibited significant anti-arthritic activity via increased SOD and CAT activity and decreased the serum level of the marker of oxidative lipid damage, malondialdehyde as well as turning -off inflammatory cascades^31^. Additionally, former research informed the Cardioprotective role of ASTA in dose (100 mg/kg) against Isoproterenol-^32^ and amiodarone^33^ induced cardiotoxicity in rats.

The improvement in cardiac rhythmicity observed in this study may be attributed to ASTA’s capacity to enhance mitochondrial function and diminish oxidative damage in cardiomyocytes, as supported by findings from previous studies^34^. Considering that oxidative stress is recognized to disrupt autonomic heart control, ASTA’s antioxidant characteristics presumably contribute significantly to the stabilization of cardiac rhythm. ASTA seems to safeguard the heart against the autonomic dysfunction commonly seen in diabetes circumstances by mitigating oxidative stress^35,36^.

Regarding contractility, ASTA’s capacity to partially restore the R amplitude in diabetic rats is consistent with other studies. ASTA has been demonstrated to enhance cardiac contractility and diminish myocardial stiffness via regulating calcium homeostasis and alleviating oxidative stress. The documented decrease in R amplitude in our study implies a comparable mechanism, suggesting that ASTA’s antioxidant characteristics may augment calcium management and boost myocardial performance in the diabetic heart^37,38^.

However, the inability of ASTA to fully normalize cardiac parameters may highlight the complexity of diabetic cardiomyopathy, which encompasses multifactorial mechanisms including fibrosis, metabolic dysregulation, and autonomic dysfunction. While ASTA effectively addresses oxidative stress and inflammation, other pathophysiological factors may limit its capacity to completely reverse diabetes-induced cardiac damage^39^. This study provides robust evidence supporting the cardioprotective effects of ASTA, particularly through its modulation of oxidative stress and myocardial contractility in diabetic models. However, further research is warranted to elucidate the mechanisms underlying its dose-dependent effects and to determine optimal dosing strategies to maximize its cardioprotective potential.

The study demonstrated that ASTA treatment significantly reduced serum markers of renal and cardiac dysfunction in diabetic rats, including urea, creatinine, CK-MB, and LDH. Particularly these reductions indicate ASTA’s protective role against diabetes-induced renal and cardiac damage, supporting previous findings that highlight the reno-protective and cardioprotective effects of ASTA through the reduction of oxidative stress and inflammation key contributors to diabetic nephropathy and cardiomyopathy^40–43^. Previous studies have reported that ASTA has been reported to attenuate oxidative stress and improve lipid and glucose metabolism in high-fat diet-induced obesity and insulin resistance models^44,45^. Moreover, its cardioprotective and nephroprotective effects have also been demonstrated in diabetic animal models, where it improved mitochondrial function, reduced inflammation, and ameliorated structural and functional impairments^12,26^. These studies collectively support our current findings and highlight the therapeutic relevance of ASTA in metabolic disorders, including diabetic complications.

The reduction in oxidative stress markers, such as MDA, alongside the restoration of antioxidant defenses like GSH in both heart and kidney tissues of diabetic rats, further underscores ASTA’s therapeutic potential in managing diabetes-related complications. The findings highlight ASTA’s efficacy in mitigating oxidative stress, demonstrating the greatest benefits, consistent with prior research^46^. ASTA is one of the most powerful antioxidant that can be protective against several kinds of oxidative damage. It is more potent than other carotenoids and vitamin E and may confer numerous health benefits^47,48^. However, further investigation is essential to refine dosing strategies and explore the underlying mechanisms in greater detail.

The intricate balance between oxidative stress and inflammation in the context of DCM and DN is crucial to understanding the pathophysiology of diabetic complications. ASTA’s ability to modulate key molecular pathways, including NOX4, Fractalkine, and the transcription factors Nrf2 and AP-1, suggests that its therapeutic potential extends beyond antioxidant properties alone. The observed upregulation of Nrf2 and AP-1 in both heart and kidney tissues underscores ASTA’s capacity to bolster endogenous defense mechanisms against oxidative stress and inflammation. Nrf2 is a master regulator of cellular redox homeostasis, controlling the transcription of various antioxidant enzymes such as HO-1, NQO1, and GCLC. These findings indicate that while ASTA exhibits potent antioxidant and anti-inflammatory effects, additional research is warranted to fully elucidate its role in regulating immune and inflammatory processes, particularly in chronic conditions such as diabetes^38,49,50^. Its activation protects against oxidative damage in diabetes by enhancing detoxification and reducing reactive oxygen species (ROS)-induced injury in organs like the heart and kidney^51,52^.

On the other hand, AP-1, a redox-sensitive transcription factor composed mainly of c-Fos and c-Jun subunits, while classically associated with inflammation and cell proliferation, also contributes to antioxidant defense. AP-1 can interact with Nrf2-regulated genes under oxidative stress conditions, reflecting a complex interplay between inflammatory and antioxidative signaling pathways^53^. Emerging evidence suggests that under pathophysiological states such as diabetes, Nrf2 and AP-1 may cooperate to orchestrate a balanced response to oxidative and inflammatory insults. For instance, AP-1 activation by oxidative stimuli can influence the expression of antioxidant enzymes like HO-1—also a downstream target of Nrf2—indicating a synergistic or compensatory relationship between these transcription factors^54^. This interplay may explain the enhanced expression of both factors in response to ASTA treatment and supports the hypothesis that ASTA’s protective effects involve coordinated modulation of overlapping antioxidant and anti-inflammatory signaling networks.

In our study, diabetic rats exhibited a significant downregulation of Nrf2 and AP-1 protein expression in both cardiac and renal tissues, indicating impaired cellular defense mechanisms and enhanced vulnerability to oxidative and inflammatory damage. These findings suggest that ASTA was able to reinstate the activity of crucial transcription factors involved in redox balance and inflammation control, thereby promoting enhanced cellular protection against diabetic complications. ASTA’s beneficial effects thus appear to arise not only from direct free radical scavenging, but also from transcriptional reprogramming that supports long-term tissue resilience and recovery in diabetic states.

Fractalkine, also known as CX3CL1, is the sole member of the CX3C chemokine family, existing in both membrane-bound and playing key roles in chemotaxis, cellular adhesion, and enhancing cell survival during hemostasis and inflammation. Recent research highlights CX3CL1 as a novel adipokine regulated by obesity and diabetes, potentially contributing to low-grade inflammation in adipose tissue linked to these conditions^55^. It facilitates monocyte adhesion to adipocytes and is upregulated in response to inflammatory cytokines. Moreover, CX3CL1 expression increases significantly in skeletal muscle cells treated with TNF-α and may be involved in diabetes-related complications like neuropathy and nephropathy^56^. Interestingly, CX3CL1 has been shown to support metabolic health, enhancing insulin secretion and preserving β-cell functionality via an MEK-dependent pathway. It has been suggested that human islets secrete CX3CL1, which protects β-cells from TNF-α-induced dysfunction by restoring critical insulin secretion pathways^57^.

Moreover, the significant modulation of Fractalkine levels by ASTA indicates its potential to mediate immune responses, which could play a crucial role in tissue repair and mitigating diabetic complications. The observed upregulation of Fractalkine following ASTA treatment may be functionally linked to reduced inflammation, as Fractalkine plays a dual role in immune modulation—facilitating leukocyte adhesion while also contributing to the resolution of inflammation^58^. This dual role is supported by previous findings showing that Fractalkine–CX3CR1 signaling helps maintain vascular integrity and modulates immune cell activity in a context-dependent manner^59^. In the setting of diabetic cardiomyopathy, the 4-fold increase in cardiac Fractalkine expression could represent a shift toward a reparative immune response, promoting regulated macrophage interaction and limiting excessive inflammatory infiltration. Such modulation may underlie the observed improvements in oxidative stress and histopathological markers. Therefore, ASTA’s ability to influence Fractalkine expression may contribute not only to its antioxidant effects but also to a broader immunomodulatory role in attenuating inflammation-related cardiac damage. These findings suggest that while ASTA demonstrates potent antioxidant and anti-inflammatory effects, further research is needed to fully elucidate its role in regulating immune and inflammatory processes, particularly in chronic conditions such as diabetes^38,60^.

In our study, the histopathological assessment of cardiac tissue showed cardiac myocardial necrosis with nuclear lysis scattered inflammatory cells and fibrosis which is considered a strong cardiac pathological change this is in agreement with^61,62^ who found the same result indicated muscle fiber disruption and inflammatory cell infiltration in the cardiac tissue of diabetic animals have been reduced in treated groups.

In the current study, the mean of glomerular diameter in diabetic rats was greater than that of non-diabetic rats indicates either normal or treated one; this is matching with Alipin et al.^63^ who confirmed that that diabetes induced glomerular enlargement in kidney, expansion of mesangial matrix and the thickening of the glomerular basement membrane are considered as initial event in development of glomerular damage. Furthermore, the Bowman’s space in kidneys of diabetic rats was wider than that of non-diabetic rats, and the percentage of proximal tubular with necrosis nuclei in diabetic rats is also greater than in the non-diabetic rats. This result is similar to the results of Kotyk et al. (2016) and Alipin et al. (2017) that reported the widening of Bowman’s space in rat upon fructose treatment^63,64^.

## Conclusion

This study demonstrates that ASTA exerts significant protective effects against diabetic cardiomyopathy and nephropathy, primarily through its antioxidant and anti-inflammatory actions. Our findings demonstrate that ASTA treatment, in dose (100 mg/kg), can significantly ameliorate the detrimental effects of diabetes on cardiac and renal function in a rat model of type 2 diabetes. These improvements evidenced modulation of serum markers of organ damage (urea, creatinine, CK-MB, and LDH) suggest that ASTA has protective effects on both cardiac and renal tissues. ASTA reduced oxidative stress and improved antioxidant defenses in cardiac and renal tissues, alongside favorable modulation of NOX4, Fractalkine, Nrf2, and AP-1, suggesting involvement of key cellular signaling pathways. Partial improvement in ECG parameters further supports ASTA’s cardioprotective role, with the 100 mg/kg dose showing the most consistent efficacy. While complete normalization of all parameters was not achieved, the observed improvements support ASTA’s therapeutic promise.

**Fig. 13:**
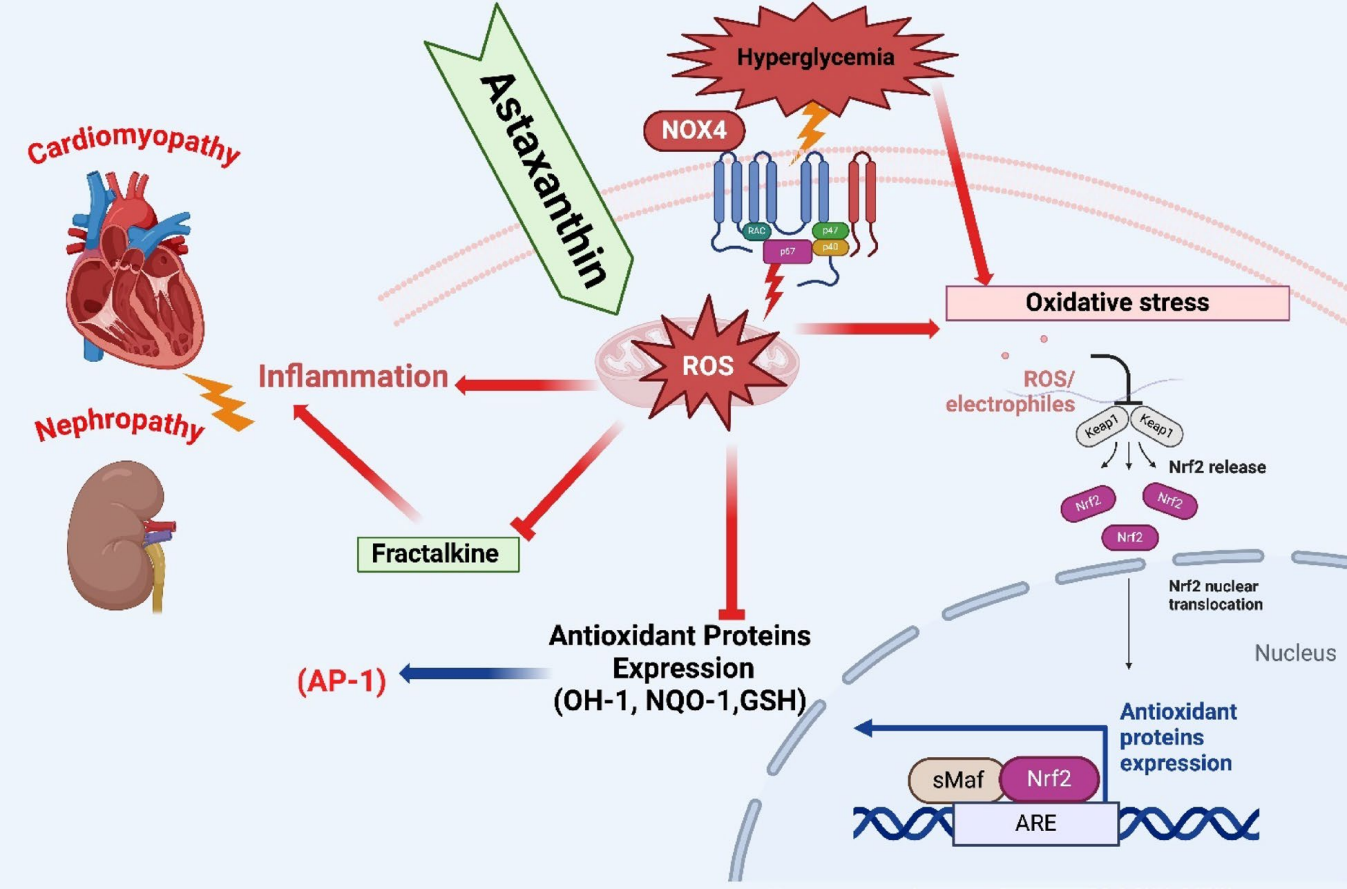
Astaxanthin exhibits its therapeutic potency against HF/HFr/STZ induced Diabetic Complications in Rats. Astaxanthin restores oxidative stress markers and boosts the expression of Nrf2 and AP-1in the heart and kidney, highlighting ASTA’s antioxidant potential, and increases Fractalkine chemokine levels, indicating anti-inflammatory effects. Overall, these findings emphasize ASTA’s combined antioxidant and anti-inflammatory actions, enhancing its therapeutic efficacy in addressing diabetic complications.

In conclusion, ASTA shows strong potential as an adjunctive therapy for diabetic complications by mitigating oxidative and inflammatory damage, as illustrated in Fig. 13, which summarizes its ability to restore redox balance and modulate signaling molecules such as Nrf2, AP-1, and Fractalkine in the heart and kidney. These findings warrant further preclinical and clinical investigation.

## Limitations of the study

While this study provides significant insights into the cardioprotective and nephroprotective effects of ASTA in diabetic rats, certain limitations must be acknowledged. First, although the study demonstrates a clear improvement in oxidative stress markers, inflammatory mediators, and functional parameters, it does not comprehensively explore the hypoglycemic mechanisms of ASTA, which warrants further investigation. Second, while molecular assays such as Western blotting and immunohistochemistry were performed to confirm protein-level changes in Nrf2 and AP-1, the mechanistic pathway involving NOX4 and Fractalkine remains partially speculative due to the absence of transcript-level analysis (e.g., qPCR). Future studies incorporating broader molecular profiling, including gene expression analysis and pathway-specific inhibitors, are needed to better delineate the full mechanistic axis through which ASTA exerts its therapeutic effects.

## Electronic supplementary material

Below is the link to the electronic supplementary material.

Supplementary Material 1

## Data Availability

All data will be available upon request to all authors.Data is provided within the manuscript or supplementary information files.
