# Supplementary material for: Astaxanthin mitigates diabetic cardiomyopathy and nephropathy in HF/HFr/STZ diabetic rats via modulating NOX4, fractalkine, Nrf2, and AP-1 pathways
Source: Sci Rep. 2025 Jun 20;15:20199. doi: 10.1038/s41598-025-06263-8 (PMC12181436; doi:10.1038/s41598-025-06263-8)
Supplement: Supplementary file 1 — Supplementary Material 1 [file 41598_2025_6263_MOESM1_ESM.pdf]

Ap-1 Heart

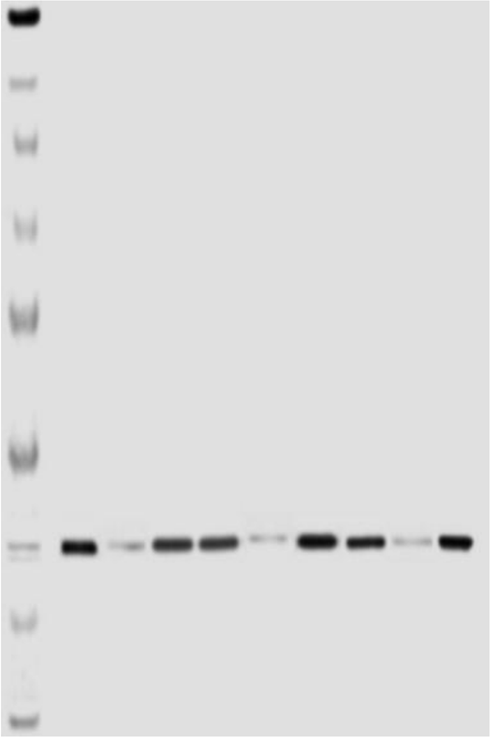

Ap-1 Kidney

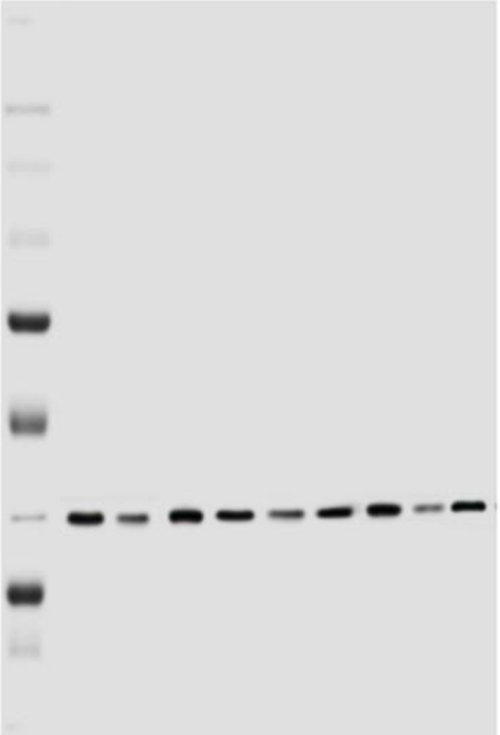

beta-actin Heart

beta-actin kidney

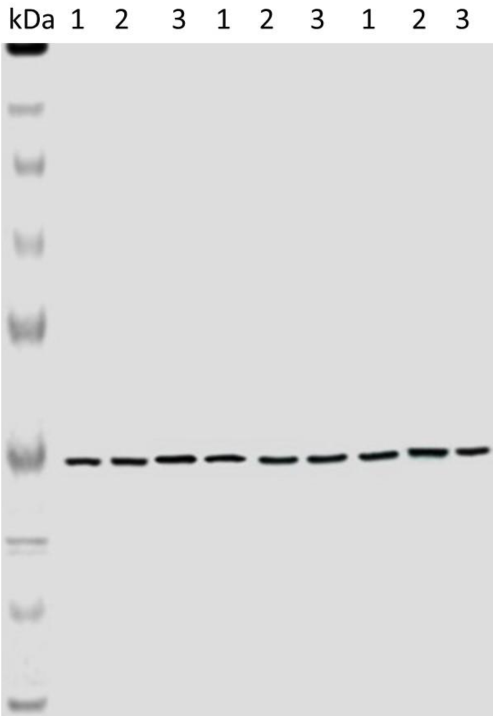
kDa 1 2 3 1 2 3 1 2 3

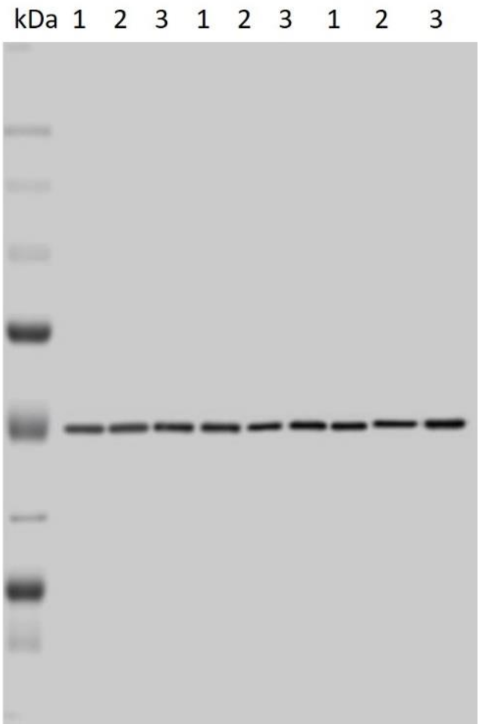
kDa 1 2 3 1 2 3 1 2 3

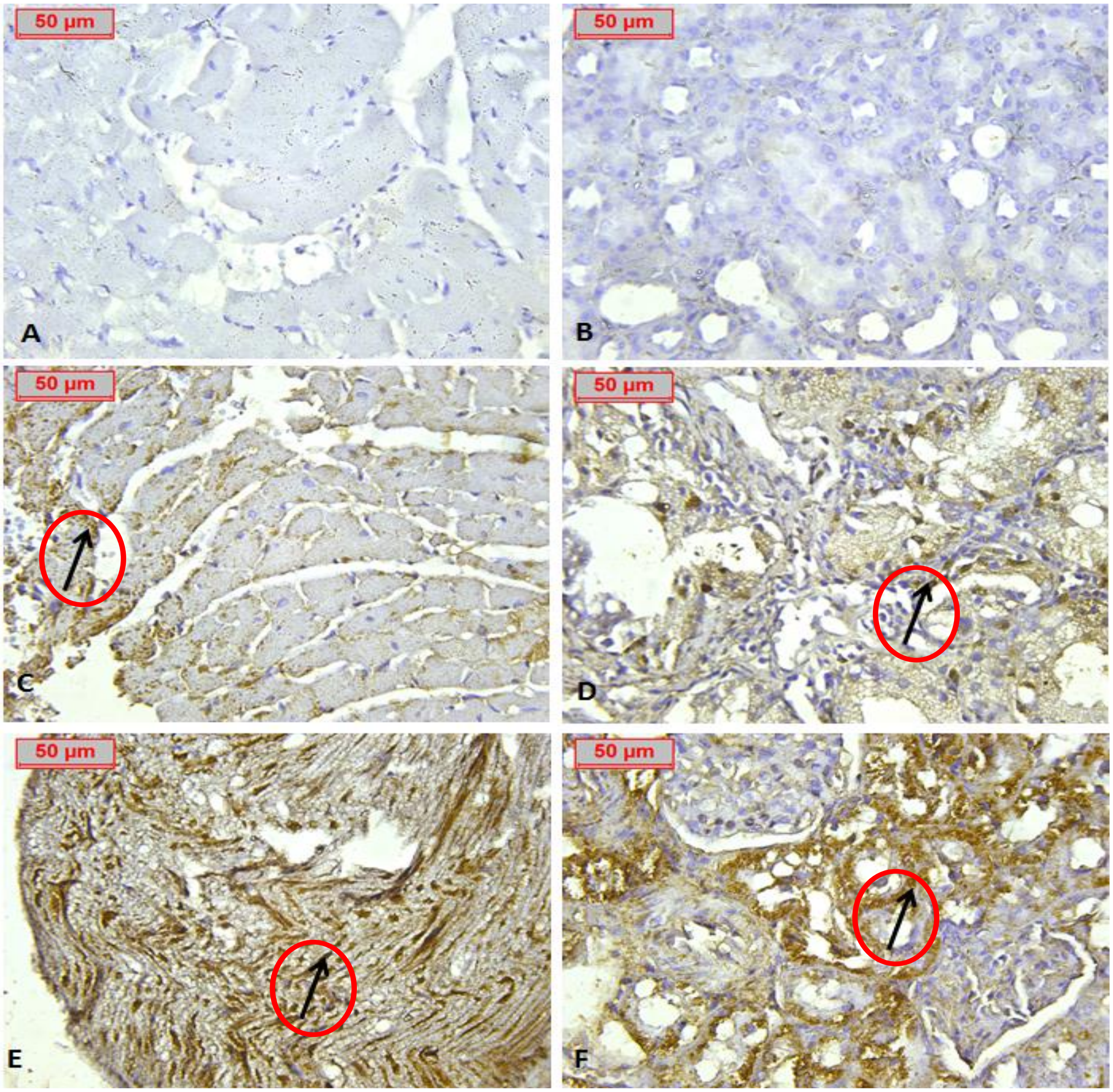
50 µm
A
50 µm
B
50 µm
C
50 µm
D
50 µm
E
50 µm
F

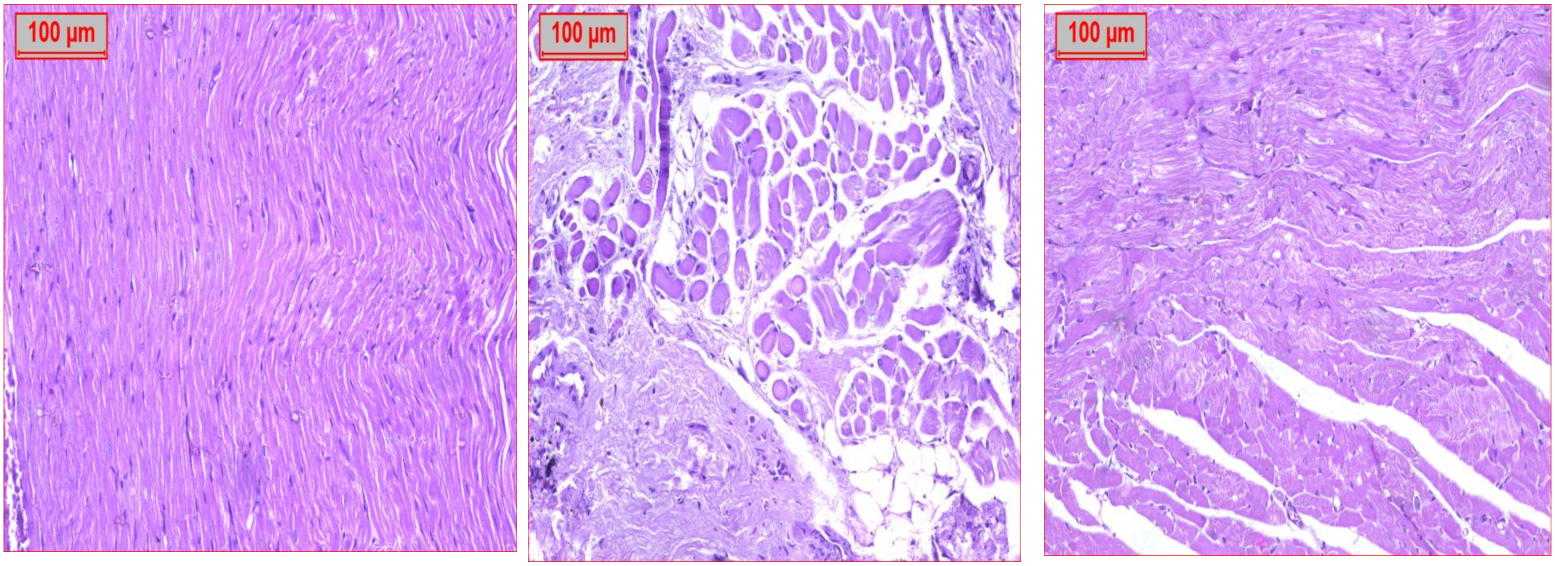

Negative Positive ASTA

Photomicrograph of different groups in cardiac tissue: control group revealed normal tissue with score 0 for both fibrosis and inflammation. Positive group revealed fibrosis with score 2 and inflammation of score 1to 2 in some areas). While Asta treated group is nearly like normal tissue except in small foci showed score 1 of inflammation.

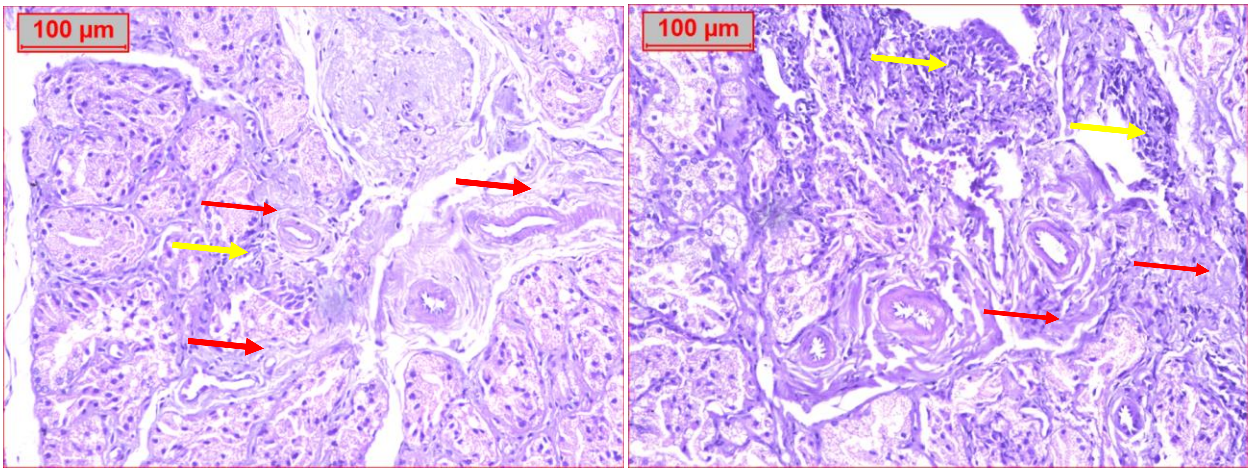

Photomicrograph of positive diabetic group revealed marked fibrosis, score 3 and inflammatory cell infiltrate; score 2

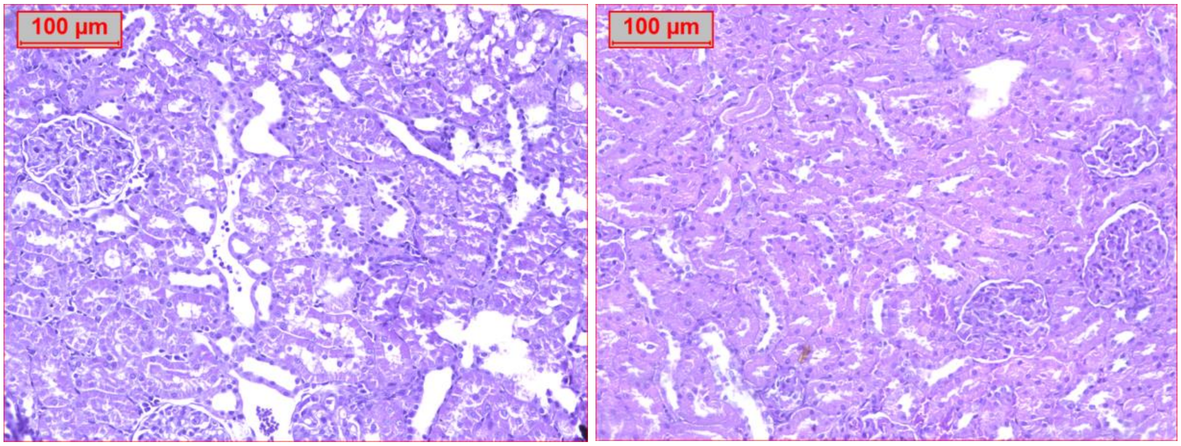

normal ASTA treated

photomicrograph revealed normal microscopic picture in both groups.
